# Engineering the methylotrophic yeast *Ogataea polymorpha* for lactate production from methanol

**DOI:** 10.3389/fbioe.2023.1223726

**Published:** 2023-06-30

**Authors:** Katrin Wefelmeier, Simone Schmitz, Anna Maria Haut, Johannes Otten, Tobias Jülich, Lars Mathias Blank

**Affiliations:** IAMB - Institute of Applied Microbiology, ABBt—Aachen Biology and Biotechnology, RWTH Aachen University, Aachen, Germany

**Keywords:** methylotrophic yeast, *Ogataea (Hansenula) polymorpha*, C1 compounds, lactate, lactate dehydrogenase

## Abstract

**Introduction:** Lactate has gained increasing attention as a platform chemical, particularly for the production of the bioplastic poly-lactic acid (PLA). While current microbial lactate production processes primarily rely on the use of sugars as carbon sources, it is possible to envision a future where lactate can be produced from sustainable, non-food substrates. Methanol could be such a potential substrate, as it can be produced by (electro)chemical hydrogenation from CO_2_.

**Methods:** In this study, the use of the methylotrophic yeast *Ogataea polymorpha* as a host organism for lactate production from methanol was explored. To enable lactate production in *Ogataea polymorpha*, four different lactate dehydrogenases were expressed under the control of the methanol-inducible MOX promoter. The L-lactate dehydrogenase of *Lactobacillus helveticus* performed well in the yeast, and the lactate production of this engineered strain could additionally be improved by conducting methanol fed-batch experiments in shake flasks. Further, the impact of different nitrogen sources and the resulting pH levels on production was examined more closely. In order to increase methanol assimilation of the lactate-producing strain, an adaptive laboratory evolution experiment was performed.

**Results and Discussion:** The growth rate of the lactate-producing strain on methanol was increased by 55%, while at the same time lactate production was preserved. The highest lactate titer of 3.8 g/L in this study was obtained by cultivating this evolved strain in a methanol fed-batch experiment in shake flasks with urea as nitrogen source. This study provides a proof of principle that *Ogataea polymorpha* is a suitable host organism for the production of lactate using methanol as carbon source. In addition, it offers guidance for the engineering of methylotrophic organisms that produce platform chemicals from CO_2_-derived substrates. With reduced land use, this technology will promote the development of a sustainable industrial biotechnology in the future.

## Introduction

Lactate is an indispensable chemical in many industries including the food and medical industry, in which it is traditionally used as an acidulant, pH regulator, or flavoring compound (Benninga, 1990). Apart from this, lactate has gained increasing importance as a building block for the production of poly-lactic acid (PLA). PLA is used as a bioplastic for various applications, including food packaging, agricultural mulch foils, and a variety of different consumer goods (Fiori, 2015). Accordingly, the lactate market reached an annual volume of US$ 3.1 billion in 2022 and is expected to grow at an annual rate of 8.0% until 2030 (Trends Analysis Report, 2022). Microbial production of lactate is especially favorable as, in contrast to chemical synthesis, enantiomerically pure lactate can be produced (Okano et al., 2010). Hence, the vast majority of lactate is produced via microbial fermentation today (Jem, van der Pol, and de Vos, 2010; Djukić-Vuković et al., 2019). Most of these industrial fermentation processes use glucose, derived from starch or sugarcane, as the use of glucose as microbial feedstock, enables high-level lactate yields (Ye et al., 2013; Djukić-Vuković et al., 2019; Ahmad, Banat, and Taher, 2020). However, using increasing amounts of glucose to replace fossil-based plastics with bio-based polymers raises ethical questions due to the potential impact on food security (Mülhaupt, 2013; Walker and Rothman, 2020; Rosenboom et al., 2022). Therefore, it is of interest to explore alternative carbon sources for microbial production processes, such as lignocellulosic waste streams or plastic waste (Wierckx et al., 2015; Wendisch et al., 2016; Karp et al., 2021). Other highly promising carbon sources are C1 molecules, i.e., molecules containing only one carbon atom. One such C1 compound is methanol (CH_3_OH, MeOH), which can be obtained from CO_2_ via (electro)chemical hydrogenation (Ganesh, 2014). If hydrogen derived from renewable energy is used for methanol synthesis, it has the potential to become an essentially unlimited and sustainable carbon source for microbial production processes (Dincer and Acar, 2015). Further, the use of methanol has the advantage that it is liquid and completely miscible with water, which greatly facilitates the fermentation process compared to gas fermentations based on other C1 compounds such as methane (CH_4_) or CO_2_ (Sahoo, Goswami, and Das, 2021). Consequently, methanol is a highly interesting feedstock for the biotechnological production of lactate, as its use could simultaneously avoid CO_2_ emissions and enable a sustainable and socially responsible production of bio-based polymers with strictly reduced land use (Cotton et al., 2020).

In this work, we explored how methylotrophic yeasts could contribute to such a vision. Methylotrophic yeasts can natively use methanol as the sole carbon and energy source. They assimilate methanol via the methanol oxidase (MOX), which catalyzes the conversion of methanol to formaldehyde (Ledeboer et al., 1985). The formaldehyde produced can then either be directed to the assimilatory or dissimilatory branch of the methanol pathway (Yurimoto, Kato, and Sakai, 2005). In the assimilatory branch, cell constituents are formed via the DHA cycle, while in the dissimilatory branch, formaldehyde is further oxidized for energy generation. This oxidation occurs via a glutathione-dependent pathway that first yields formate and eventually CO_2_, producing NADH as reducing equivalent (Yurimoto and Sakai, 2019).

The methylotrophic yeast used in this study is *O. polymorpha* (formerly *Hansenula polymorpha* or *Pichia angusta*). Apart from its methylotrophy, it has several advantageous characteristics, such as its tolerance to a wide pH range, growth to high cell densities, and strong methanol-inducible promoters (Cox et al., 2000; Manfrão-Netto et al., 2019; Zhai et al., 2021). Particularly, its ability to tolerate temperatures of up to 50 °C makes it a promising host organism for producing methanol-derived chemicals (Cabeç-Silva and Madeira-Lopes, 1984). The assimilation of methanol in yeasts requires oxygen as an electron sink, which leads to a substantial production of heat (Cotton et al., 2020). Therefore, *O. polymorpha*’s thermotolerance could be a great asset to reduce the cooling costs in an industrial process for platform chemicals derived from methanol. *O. polymorpha* has already been applied extensively as a production host for recombinant proteins, e.g., for the production of hepatitis B vaccines (Seo et al., 2008), phytase (Mayer et al., 1999) or interferons (Müller II et al., 2002). In contrast, the number of studies using *O. polymorpha* to produce low molecular weight biochemicals is still limited. Eilert et al. produced the compatible solute 5-hydroxyectoine by introducing a heterologous production pathway into *O. polymorpha* (Eilert et al., 2013)*.* Further, overproduction of free fatty acids (Gao et al., 2022) and the terpenoid β-elemene (Ye et al., 2023) has recently been demonstrated in *O. polymorpha*. Apart from these examples, the use of *O. polymorpha* as a beneficial production host for low molecular weight biochemicals and specifically its potential for methanol-based production processes is still largely unexplored.

In this study, we want to exploit the native ability of *O. polymorpha* for methanol assimilation to produce lactate through the expression of heterologous lactate dehydrogenases. We, therefore, developed *O. polymorpha* strains that overproduce heterologous lactate dehydrogenases and characterized the strains in microtiter plate and shake flask cultivations. We also adjusted the composition of the cultivation medium and performed an adaptive laboratory evolution experiment to enhance growth of the lactate-producing *O. polymorpha* strains on methanol as carbon source.

## Materials and methods

### Strains and media

The strain *O. polymorpha* NCYC495 *leu1.1* sequenced by Riley et al. (Riley et al., 2016) serves as a basis for all *O. polymorpha* strains in this study. In order to enable genetic engineering of this strain through CRISPR-Cas9 editing, an Improved-Cas9 (iCas9) was genomically integrated into the *MET2* gene of *O. polymorpha*, which makes the strain methionine auxotrophic. iCas9 is based on the Cas9 enzyme of *Streptococcus pyogenes* and has two point mutations leading to a higher cleavage efficiency compared to the non-mutated version (Bao et al., 2015). For pre-cultures, the yeast cells were grown at 37 °C in a rich YPD-medium (1% yeast extract, 2% peptone, 2% glucose). For growth characterization of the engineered strains, the defined mineral Verduyn medium (Verduyn et al., 1992) was used, supplemented with varying concentrations of methanol (0.5%–2% (v/v)) as carbon source. The medium was either buffered with 100 mM MES for cultivation of the ALE_Lh_LDH strain with urea as nitrogen source (N source) or with 100 mM of KH-phthalate for all residual cultivations. As required, the medium was further supplemented with 0.5 g/L leucine, 20 mg/L histidine, 20 mg/L methionine, 20 mg/L adenine, or 1 g/L of yeast extract. Originally, Verduyn medium contains 5 g/L (NH_4_)_2_SO_4_, which corresponds to 1.06 g_Nitrogen_/L. For the nitrogen source variation experiments, the ammonium sulfate was replaced by either urea, ammonium nitrate, yeast extract (Carl Roth, Karlsruhe, Germany; total nitrogen content: 8%), or peptone (Carl Roth, total nitrogen content 10%). The total amount of nitrogen in the media was adjusted for each N source to correspond to the original amount in the Verduyn medium.

For plasmid amplification, *Escherichia coli* cells (NEB^®^ 10-beta, High Efficiency) were used, which were grown at 37 °C in lysogeny broth (LB) containing 100 μg/mL ampicillin for selection.

### Strain construction

For genomic integration of the lactate dehydrogenases, repair fragments were constructed containing the LDH gene under the control of the pMOX promoter and the tMOX terminator. This expression cassette was framed by two 1 kb sequences homologous to the upstream and downstream regions of the *HIS2* gene of *O. polymorpha*. The LDH coding sequences were codon-optimized for *O. polymorpha* and ordered as synthetic DNA fragments via the GeneArt gene synthesis service (Thermo Fisher Scientific, Waltham, MA, United States ). The genetic parts for the repair fragments were amplified using the Q5^®^ High-Fidelity 2X Master Mix (NEB, Ipswich, MA, United States ) and then assembled into a linear DNA fragment using splicing by overlap extension PCR (SOE-PCR). Integration of the repair fragments into the genome was performed using CRISPR/Cas9 as described by Wang et al. (Wang et al., 2018). The *JEN1* transporter gene of *Saccharomyces cerevisiae* was expressed under the control of the constitutive pTEF1 promoter and the tAMO terminator. It was codon-optimized and integrated into the promoter region of the *TEF1* gene in *O. polymorpha* using a pHIP expression plasmid conferring Zeocin resistance (Saraya et al., 2012). Transformation of the genetic parts into *O. polymorpha* was performed through LiAc/single-stranded carrier DNA/PEG transformation (Holkenbrink et al., 2018). After transformation, the cells were selected on YPD agar plates with 100 μg/L nourseothricin or Zeocin. Correct integration of the LDH and JEN1 repair fragments was then verified through colony-PCR with the Phire Plant Direct PCR Master Mix (Thermo Fisher Scientific). For colony-PCR, one primer targeting the integrated cassette was used, while the other one bound to the surrounding genomic region.

### Cultivation conditions

Microbial cultivations in this work were either performed in 500 mL shake flasks or microtiter plates. For microtiter plate cultivations, the BioLector system (Beckman Coulter Life Sciences, Indianapolis, IN, United States) was applied. Cultivations of *O. polymorpha* in the BioLector were performed in 48-well clear bottom FlowerPlates^®^ (Beckman Coulter Life Sciences), covered with an adhesive gas-permeable membrane (Beckman Coulter Life Sciences). Each well was filled with 1 mL of Verduyn medium and inoculated to a starting optical density of 0.2 at 600 nm (OD_600 nm_). Plates were then cultivated at 37 °C with a shaking frequency of 1,000 rpm. The accumulation of biomass in the cultures was tracked online by measuring the scattered light in each well at 620 nm (Gain 40). All shake flasks experiments were performed in 500 mL shake flasks filled with 50 mL of Verduyn medium. Flasks were cultivated at 37 °C and 250 rpm shaking. All experiments were performed in biological triplicates, if not otherwise stated.

### Analytical methods

Consumption of the carbon source methanol and production of lactate was monitored using high-performance liquid chromatography (HPLC-UV-RI). To this end, 1 mL of culture was centrifuged at maximum speed and the supernatant was filtered with Rotilabo syringe filters (Carl Roth, pore size 0.2 µm). The filtered supernatants were subsequently analyzed with a DIONEX UltiMate 3000 HPLC System (Thermo Fisher Scientific) equipped with a Metab-AAC column (300 × 7.8 mm, ISERA, Düren, Germany). For elution 5 mM H_2_SO_4_ at a flow rate of 0.6 mL/min and a temperature of 60 °C was used. Detection was performed with a SHODEX RI-101 detector (Showa Denko Europe GmbH, München, Germany) and a DIONEX UltiMate 3000 Variable Wavelength Detector (Thermo Fisher Scientific) set to 210 nm.

### Adaptive laboratory evolution

To improve the growth behavior of the lactate-producing strains on methanol as the sole carbon source, the strains were cultivated in 24-well System Duetz microtiter plates (Kuhner Shaker, Birsfelden, Switzerland) on Verduyn medium containing 2% methanol (v/v). Once the strains reached early exponential growth phase 50 µL were transferred to a fresh well with Verduyn medium. In total each strain was transferred 25 times to a new well. After 25 transfers, single colonies of the evolved populations were isolated on YPD agar plates. Growth characterization was then performed in BioLector microtiter plate experiments (*cf.* section *Cultivation conditions*) and lactate titers were determined at the end of the cultivation using HPLC analysis (*cf.* section *Analytical methods*). To determine the growth rate of the evolved colonies they were cultivated in 500 mL shake flasks equipped with the Cell Growth Quantifier (CGQ; SBI Scientific Bioprocessing, Baesweiler, Germany), which allows a continuous online biomass measurement by light scattering.

### Whole genome sequencing

For genomic DNA extraction in *O. polymorpha,* the YeaStar Genomic DNA Kit (Zymo Research, Freiburg, Germany) was used. The isolated genomic DNA was sent for whole genome sequencing and SNP identification to IIT GmbH (Bielefeld, Germany). The NCBI’s Basic Local Alignment Search Tool (BLAST (Altschul et al., 1997)) was used to perform sequence similarity analyses against the NR protein database. To predict the protein structure of the methanol oxidase the AlphaFold Monomer v2.0 pipeline was used (Jumper et al., 2021; Varadi et al., 2022).

## Results

### Choice of lactate dehydrogenase

Lactate is synthesized from pyruvate in an NAD^+^ regenerating reaction catalyzed by the enzyme lactate dehydrogenase (LDH). It has been shown that the choice of LDH plays a crucial role in establishing high-level lactate production in a heterologous production host (Branduardi et al., 2006; Ilmén et al., 2013). For engineering lactate production in *O. polymorpha*, we selected 4 LDH genes originated from *Bos taurus*, *Leuconostoc mesenteroides*, *Lactobacillus helveticus*, and *Lactiplantibacillus plantarum*. All of the selected genes have already been successfully overexpressed for lactate production in different yeast species (Table 1). A mutated gene variant was chosen for the LDH gene of *L. plantarum.* The mutation leads to the replacement of an aspartate residue with a glycine residue at position 94 of the amino acid chain. This amino acid substitution was reported to lead to increased lactate titers and productivities compared to the wildtype variant when expressed in *S. cerevisiae* (Branduardi et al., 2006)*.*

**TABLE 1:** Tested LDH candidate genes and their properties.

Origin species	Produced enantiomer	K_m_ value (pyruvate)	K_cat_ value (pyruvate)	Heterologous expression in yeast
*Bos taurus*	L-Lactate	0.13 mM (Ishida et al., 2005)	2.0 s^-1^ (Ishida et al., 2005)	(Ishida et al., 2005)
*Leuconostoc mesenteroides*	D-Lactate	0.58 mM (Li et al., 2012)	2900 s^-1^ (Li et al., 2012)	(Yamada et al., 2019)
*Lactobacillus helveticus*	L-Lactate	0.25 mM (Savijoki and Palva, 1997)	643 s^-1^ (Savijoki and Palva, 1997)	(Ilmén et al., 2013)
*Lactiplantibacillus plantarum* Amino acid variant: D94G	L-Lactate	1.1 mM (Branduardi et al., 2006)	5.6 s^-1^ (Branduardi et al., 2006)	(Branduardi et al., 2006)

**FIGURE 1:**
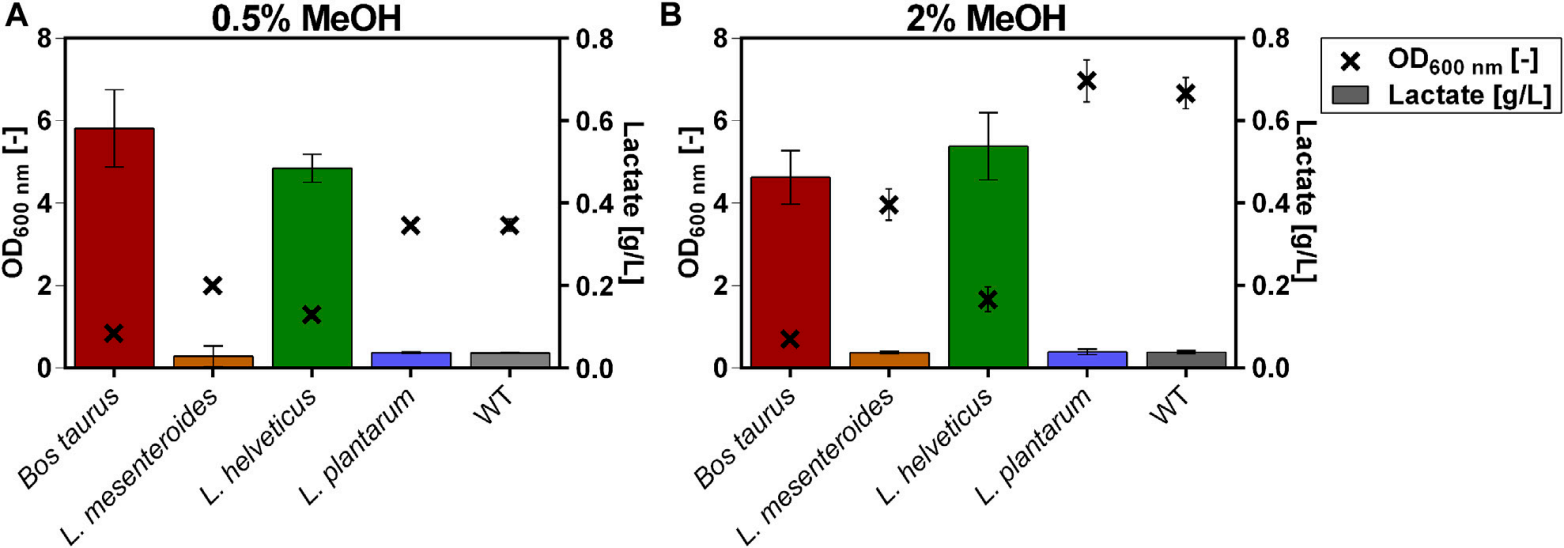
Lactate production of *O. polymorpha* strains overexpressing heterologous lactate dehydrogenase genes from *Bos taurus*, *Leuconostoc mesenteroides*, *Lactobacillus helveticus* or *Lactiplantibacillus plantarum*. Cultivations were performed in a BioLector microtiter plate cultivation experiment with **(A)** 0.5% (v/v) methanol (MeOH) or **(B)** 2% (v/v) MeOH as carbon source. Lactate titers and end OD were measured after a cultivation time of 7 days. Error bars represent the standard deviation of three biological replicates.

The LDH genes were codon-optimized for *O. polymorpha* and expressed using the methanol-inducible MOX promoter and the strong MOX terminator. Figure 1 shows lactate production and final optical densities (ODs) resulting from the expression of the LDHs in *O. polymorpha* when using 0.5% or 2% (v/v) methanol as carbon source in microtiter plate batch cultivations. As a control, the initial strain without LDH integration (WT) was included in the experiment. The cells were cultivated in Verduyn-medium for 7 days (Figure 1). Yeast extract was added as an additional nitrogen source and auxiliary substrate, as the engineered strains showed considerably reduced growth compared to the unmodified WT strain. No significant lactate production was observed in both the strain overexpressing the LDH gene of *Leuconostoc mesenteroides* (Lm_LDH) and the LDH gene of *Lactiplantibacillus plantarum* (Lp_LDH). For the two remaining strains with the *Bos taurus* LDH (Bt_LDH) and the *Lactobacillus helveticus* LDH (Lh_LDH) a drastically elevated lactate production was observed. Using 0.5% MeOH as carbon source in the cultivation medium, the Bt_LDH strain produced 0.6 ± 0.1 g/L lactate, while the Lh_LDH strain produced 0.5 ± 0.0 g/L lactate (Figure 1A). Apart from the lactate peak, no potential by-products such as ethanol or pyruvate were observed in the HPLC chromatograms (data not shown). While the Lh_LDH and Bt_LDH strain produced lactate, these two strains showed significantly impaired growth. The WT strain reached a final OD of 3.5 ± 0.2, whereas the Bt_LDH and Lh_LDH strain only reached final optical densities of 0.8 ± 0.0 and 1.3 ± 0.1, respectively (Figure 1A). When a higher substrate concentration of 2% MeOH was used, lactate titers did not differ considerably (Figure 1B). Both, the Bt_LDH and Lh_LDH strain produced 0.5 ± 0.1 g/L lactate. Again, no increased lactate production was observed for the Lm_LDH and the Lp_LDH strain. With this higher methanol concentration an even more severe growth defect of the lactate-producing strains compared to the WT could be noticed. While the WT strain reached an OD of 6.7 ± 0.4, the Lh_LDH strain only reached an OD of 1.7 ± 0.3 and the Bt_LDH grew even less and only reached an OD of 0.7 ± 0.1 (Figure 1B). It became clear that adding higher methanol concentrations than the initially tested 0.5%, did not automatically lead to higher lactate titers. There is an obvious trade-off between cell growth and lactate production, as the non-producing strains could reach significantly higher cell densities compared to the two strains overproducing lactate. To exclude that this effect is the reason of cell stress resulting from low pH-levels due to the lactate being produced, the end-pH after the cultivation was measured (Supplementary Material: Supplementary Figure S1). However, the pH at the end of the cultivation did not differ drastically between the lactate-producing and non-producing cultures and remained close to the initial pH value of 5 for all tested strains and replicates. Accordingly, it was concluded that it is not possible to increase lactate titers simply by increasing the initial amount of methanol in the culture.

### Lactate utilization

**FIGURE 2:**
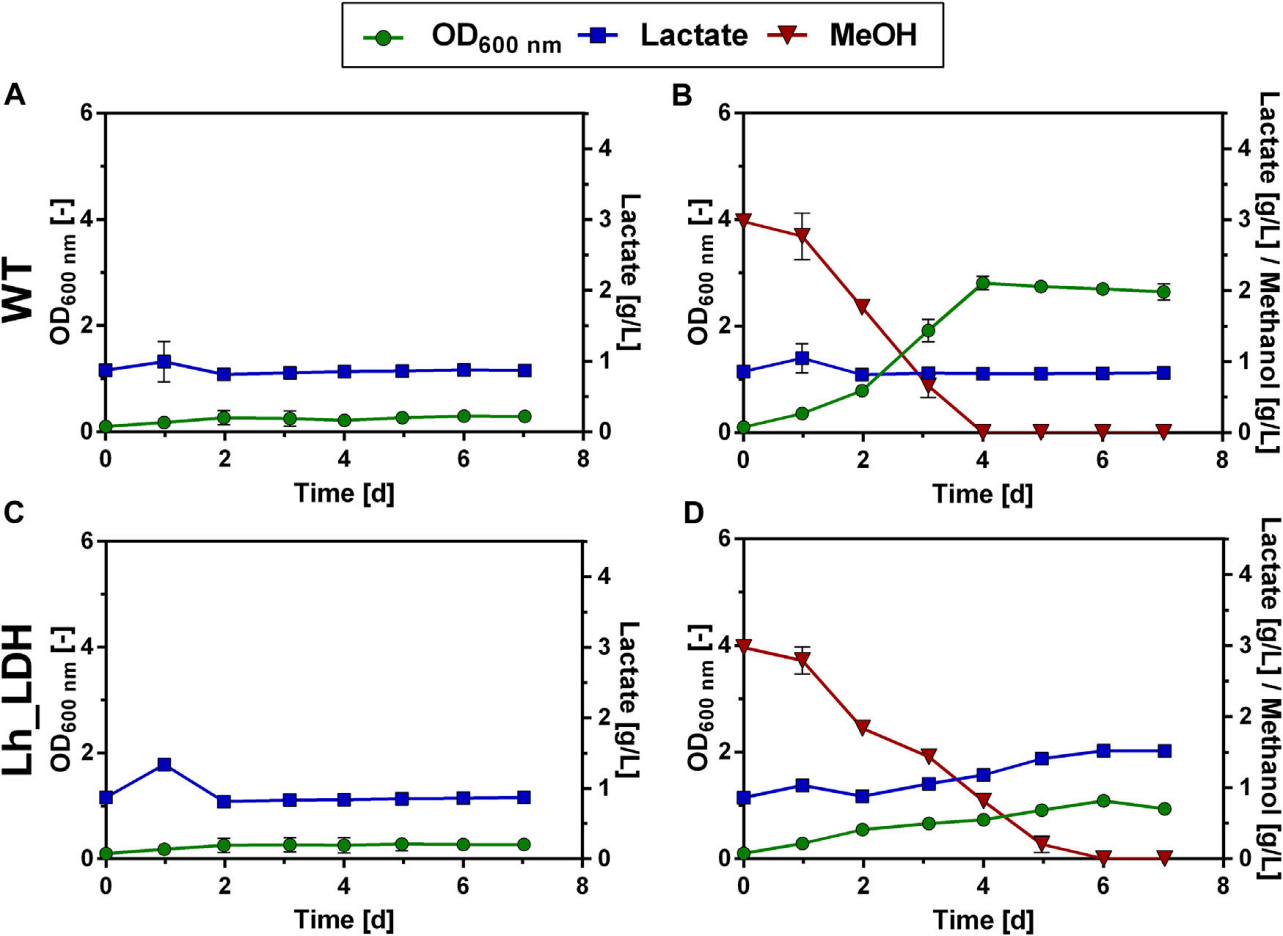
Lactate consumption and growth of *O. polymorpha* WT **(A,B)**, Lh_LDH **(C,D)** in Verduyn medium with 1 g/L lactate **(A,C)** and 1 g/L lactate +0.5% methanol **(B,D)** as carbon sources. Cultivation in microtiter plates for 7 days. In all graphs, the standard deviation is represented by error bars from three biological replicates.

In order to establish production of a heterologous product in a microorganism it is critical to consider transport mechanisms in and out of the cell (Vickers, Blank, and Krömer, 2010). To assess the uptake of lactate, it was tested, whether the *O. polymorpha* WT strain and the Lh_LDH strain could grow on 1 g/L of L-lactate supplied to the cultivation medium (Figures 2A,C). The same experiment was repeated with an additional 0.5% methanol present in the culture next to the lactate (Figures 2B,D). Neither the WT strain nor the Lh_LDH were able to take up the lactate from the medium and use it for growth under these cultivation conditions (Figures 2A,C). When methanol was additionally supplied to the cultures (Figures 2B,D), methanol was consumed from the medium and both strains grew, but neither strain consumed the lactate from the culture, even after methanol was depleted. As expected, for the Lh_LDH strain an increase in the lactate titer was observed (Figure 2D), as the addition of methanol induces the expression of the LDH gene and thus enables lactate production. To exclude that the used lactate concentration of 1 g/L was too low for the endogenous lactate uptake system of *O. polymorpha* the Lh_LDH strain was additionally cultivated with 2.5 g/L and 5 g/L of lactate and 0.5% methanol (Supplementary Material: Supplementary Figure S2). Also under these conditions, methanol was completely consumed from the culture, whereas no uptake of lactate was observed.

**FIGURE 3:**
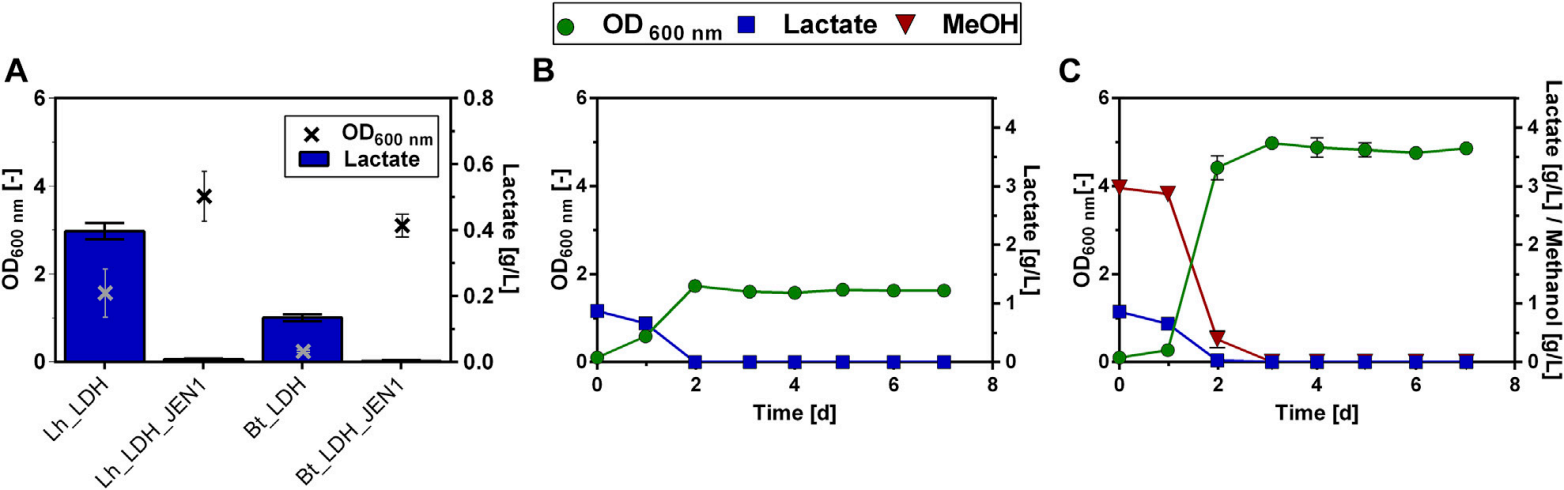
Lactate production and consumption by *O. polymorpha* strains producing the Jen1 lactate permease from *S. cerevisiae*. Final optical density and lactate titer of Lh_LDH and Bt_LDH compared to the same strains expressing *JEN1* (Lh_LDH_JEN1, Bt_LDH_JEN1) in a microtiter plate cultivation with 0.5% MeOH as carbon source **(A)**. Growth and Consumption of 1 g/L supplied lactate **(B)** or 1 g/L supplied lactate and 0.5% MeOH **(C)** by Lh_LDH_JEN1 strain in shake flask cultivations. All error bars represent the standard deviation of three biological replicates.

Apart from monitoring the uptake of lactate, we also examined the export of lactate. Lactate has a pK_a_ value of 3.86. Consequently, at the neutral intracellular pH of the yeast, lactate dissociates to its deprotonated form. Since deprotonated acids can generally not diffuse through the plasma membrane, they must be actively transported out of the cell. Branduardi et al. reported that by facilitating the export of lactate in *S. cerevisiae* (*S. cerevisiae*) through the coexpression of an LDH and the native lactate permease Jen1, lactate production could be increased, compared to only expressing the LDH alone (Branduardi et al., 2006). Therefore, the *JEN1* gene from *S. cerevisiae* was introduced into the lactate-producing *O. polymorpha* strains under the control of the constitutive TEF1 promoter, creating the strains Lh_LDH_JEN1 & Bt_LDH_JEN1. Both strains were cultivated in a microtiter batch cultivation with methanol as the carbon source and compared to the respective parental strain without the Jen1 transporter (Figure 3A). Surprisingly, it was seen that lactate production was almost completely abolished in both strains expressing the *JEN1* gene (Figure 3A). Instead, the Jen1-strains were able to take up and grow on externally supplied lactate, both in the presence and absence of additional methanol in the culture (Figures 3B,C). Consequently, it was concluded that the expression of Jen1 did not remove any limitations in lactate export as expected, but instead enables the efficient uptake of lactate from the medium and its utilization as carbon source.

### Fed-batch cultivation mode

**FIGURE 4:**
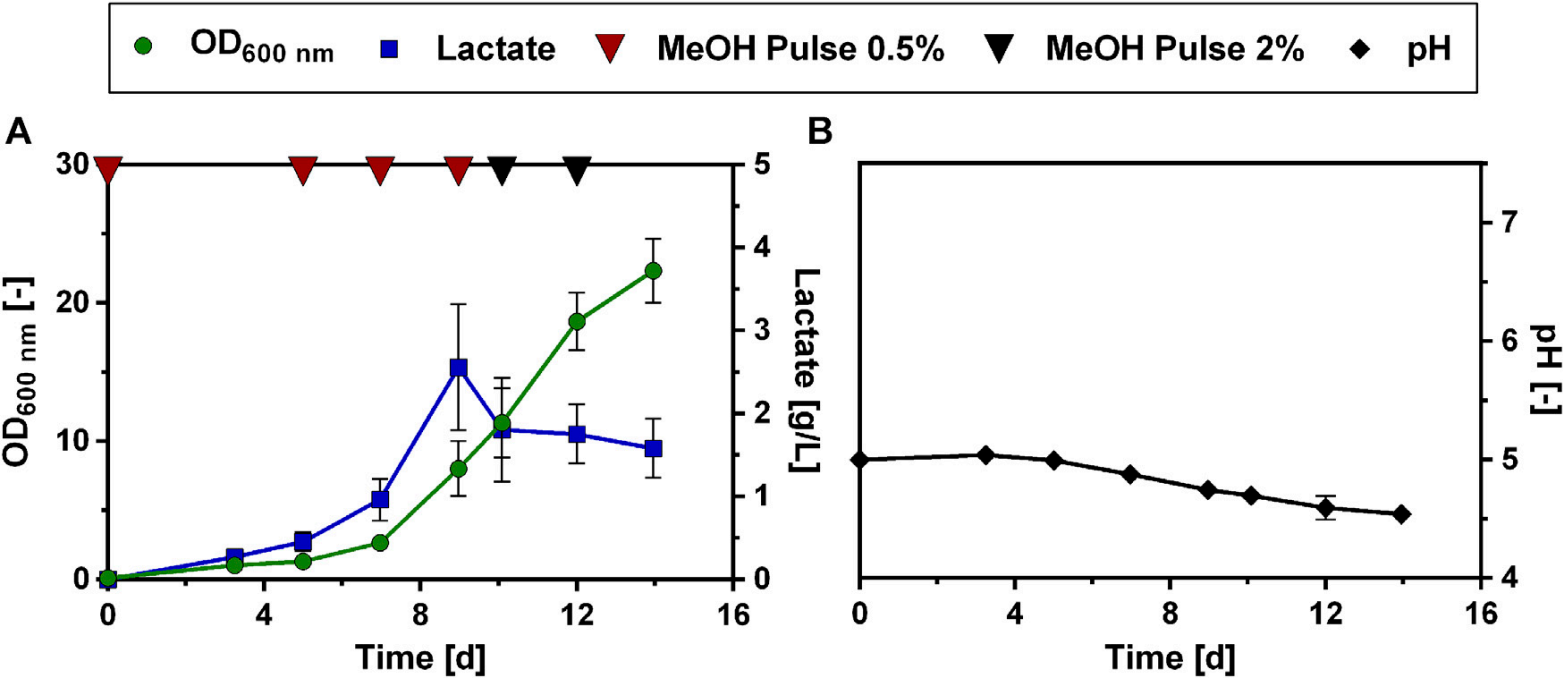
Cultivation of *O. polymorpha* Lh_LDH in a methanol fed-batch experiment. Development of lactate titer (**A**, blue squares), optical density (**A**, OD_600 nm_, green dots) and pH (**B**, black dots) cultivating an *O. polymorpha* strain overexpressing Lh_LDH in Verduyn medium with 0.5% MeOH as initial carbon source. Additional pulses of 0.5% or 2% methanol (**A**, red and black triangles, respectively) were supplied to the culture upon methanol consumption. Experiments were performed in 500 mL shake flasks. Error bars represent the standard deviation of three biological replicates.

Since the introduction of a lactate transporter did not have the desired effect of increasing lactate production, we further investigated how to enhance lactate production by modifying the cultivation mode. To circumvent methanol toxicity and at the same time increase lactate production, a two-stage cultivation, divided into a biomass production and a product formation phase was tested as an option. This cultivation exploits the fact that the LDH genes are under the control of the methanol-inducible MOX promoter. Therefore, it was considered to first use glucose or glycerol as carbon source for biomass accumulation and then induce lactate production by the addition of methanol to the culture in late exponential phase. Different combinations of glucose, glycerol and methanol as carbon sources were tested in a microtiter plate cultivation experiment with the Lh_LDH strain (Supplementary Material: Supplementary Figure S3). While biomass densities were greatly enhanced using this strategy, none of the substrate combinations yielded a higher lactate titer compared to using only 0.5% or 2% methanol as the sole carbon source (Supplementary Material: Supplementary Figure S3). Therefore, the idea to use glucose or glycerol as a co-substrate for enhanced biomass formation was not pursued further. Instead, it was investigated whether lactate production titers could be enhanced through feeding multiple methanol pulses in fed-batch experiments in shake flasks. In these experiments, the strains were cultivated in 500 mL shake flasks with an initial methanol concentration of 0.5%. Regular samples were taken and methanol consumption was monitored using HPLC analysis. Upon methanol consumption, the cultures were fed with another pulse of 0.5% methanol. The rate at which methanol was taken up increased during cultivation. Hence, the cultures were fed with 2% methanol as soon as the 0.5% methanol was consumed in less than 48 h, to avoid methanol starvation of the cells. Figure 4 shows the development of the lactate concentration and OD over the course of this cultivation with the Lh_LDH strain. The use of this fed-batch mode of cultivation improved lactate production of the Lh_LDH strain considerably. Comparable to batch cultivation of the Lh_LDH strain (Figure 1) the initial methanol concentration of 0.5% resulted in a lactate titer of 0.5 ± 0.1 g/L (Figure 4A). The initially supplied methanol was consumed after 5 days, after which a second pulse was added to the cultures. After that pulse, the rate of lactate production as well as growth increased, and a maximum lactate titer of 2.6 ± 0.5 g/L was obtained. This lactate concentration was reached on day 9 of the cultivation, after which the lactate concentration in the culture decreased to a value of 1.6 ± 0.4 g/L by day 14, even though the cells continued to grow and were supplied with further methanol pulses (Figure 4A). The pH during the cultivation dropped from its initial value of 5 to a value of 4.5 ± 0.1 at the end of the cultivation (Figure 4B).

A corresponding experiment was also carried out with the Bt_LDH strain (Supplementary Material: Supplementary Figure S4). However, this strain consumed the added methanol more slowly than the Lh_LDH strain and did not show improved lactate production compared to the batch experiment, even after multiple added methanol pulses. Therefore, it was decided to focus on optimizing lactate production for the Lh_LDH strain, as this strain performed better. Both the Lh_LDH strain and Bt_LDH strain were subsequently send for whole genome sequencing (data not shown). The Lh_LDH strain was found to carry one copy of the LDH expression cassette, whereas the Bt_LDH strain carried three consecutively integrated copies. Even though *O. polymorpha* is known to stably replicate numerous copies of genes integrated in its genome (Agaphonov et al., 1999; Terentiev et al., 2004), this increased copy number might be one of the reasons, why the Bt_LDH strain showed considerably poorer growth on methanol, as the expression of multiple cassettes with the strong methanol-inducible MOX promoter, most likely drains considerable resources from cell growth.

### Impact of nitrogen sources

**FIGURE 5:**
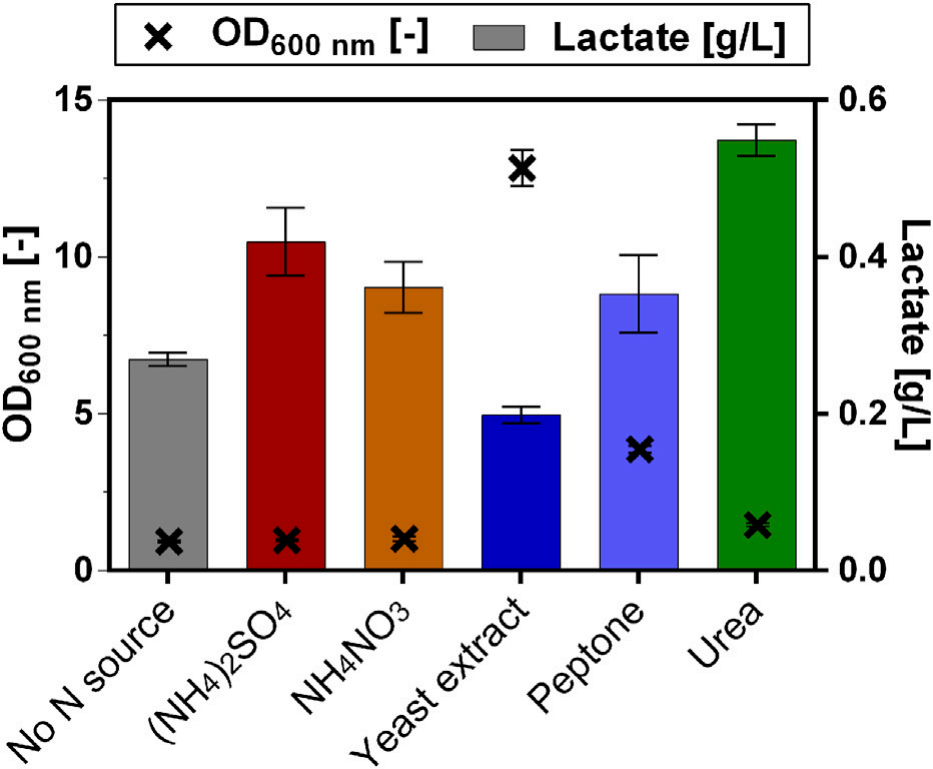
Lactate production with varying N sources. Lactate production (bars) and final optical density (OD_600,_ crosses) of *O. polymorpha* strain overexpressing Lh_LDH cultivated in Verduyn medium with 0.5% MeOH as carbon source and different nitrogen sources. The original Verduyn medium contained (NH_4_)_2_SO_4_ (ammonium sulfate) as N source. As alternative nitrogen sources NH_4_NO_3_ (ammonium nitrate), yeast extract, peptone and urea were tested. As a control, Verduyn medium without additional N source was included (No N source). Experiments were performed in a System Duetz microtiter plate, in which strains were cultivated for 7 days. Error bars represent the standard deviation of three biological replicates.

As it has been shown that the choice of nitrogen source (N source) can have a significant impact on carboxylic acid production in different fungi species (Zhang, Jin, and Kelly, 2007; Yuwa-Amompitak and Chookietwattana, 2014; Guo et al., 2021) we examined whether this would also be the case for *O. polymorpha*. The original N source in the chosen Verduyn medium is ammonium sulfate ((NH_4_)_2_SO_4_) (Verduyn et al., 1992). Additionally, 1 g/L of yeast extract was used as auxiliary substrate in the cultivation medium and since the strain has multiple auxotrophies there are further amino acids supplemented (*cf. Material & Methods* section). The yeast extract and the amino acids thus represent additional available N sources for the lactate-producing *O. polymorpha* strains but are essential for growth and thus cannot be substituted. Consequently, only ammonium sulfate was replaced as N source in the Verduyn medium. As alternative nitrogen sources we chose ammonium-nitrate (NH_4_NO_3_), yeast extract (Carl Roth GmbH, total nitrogen content: 8%), peptone (Carl Roth GmbH, total nitrogen content 10%) and urea (CO(NH_2_)_2_), which were compared to the original medium with ammonium sulfate (Figure 5). To ensure comparability, the total concentration of nitrogen in the media for each N source was adjusted to match the original amount in the Verduyn medium (5 g/L of ammonium sulfate = 1.06 g/L nitrogen). Additionally, Verduyn medium containing no additional N source and only the supplemented amino acids and yeast extract was included in the experiment as a control. The different cultures differed noticeably in their lactate production. The control with the unmodified Verduyn medium produced 0.4 ± 0.0 g/L of lactate from the 0.5% of fed methanol (Figure 5). Surprisingly, neither the culture with ammonium nitrate (0.4 ± 0.0 g/L), yeast extract (0.2 ± 0.0 g/L), peptone (0.4 ± 0.0 g/L), nor the culture without any additional N source (0.3 ± 0.0 g/L) achieved a higher lactate titer. Only the culture, in which urea was used as N source showed an increased lactate production (0.6 ± 0.0 g/L).

**FIGURE 6:**
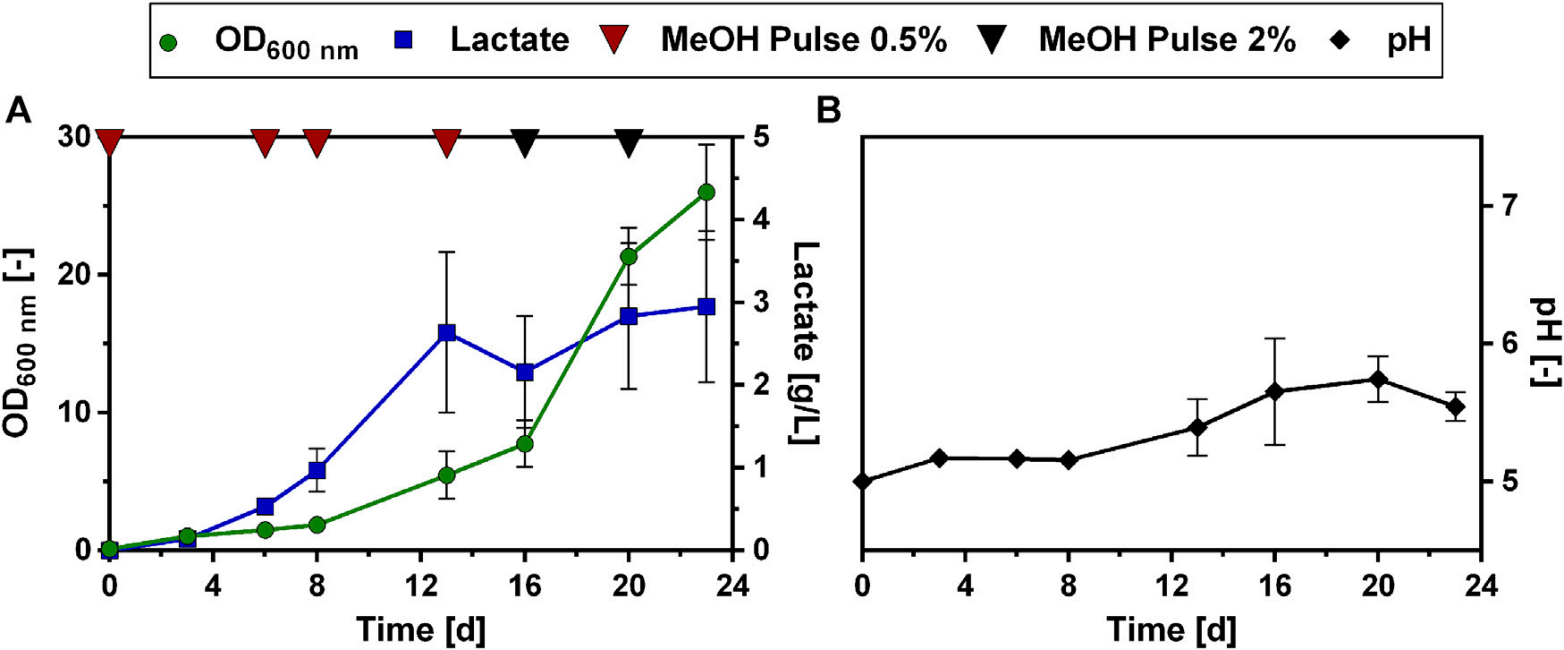
Methanol fed-batch with urea as N source. Development of lactate titer (**A**, blue squares), optical density (**A**, OD_600 nm_, green dots), methanol concentration (**B**, red dots) and pH (**C**, black dots) cultivating an *O. polymorpha* strain overexpressing Lh_LDH cultivated in Verduyn medium with urea as N source and 0.5% MeOH as initial carbon source. Additional pulses of 0.5% or 2% methanol (**A**, red and black triangles, respectively) were supplied to the culture upon methanol consumption. Experiments were performed in 500 mL shake flasks. Error bars represent the standard deviation of three biological replicates.

To elucidate whether this positive effect of urea as N source could also be reproduced in a fed-batch cultivation mode, the modified Verduyn medium with urea as N source was then tested in a methanol fed-batch experiment, analogous to the experiment described in Figure 4. The Lh_LDH strain was cultivated in 500 mL shake flasks with an initial methanol concentration of 0.5% and then fed with additional pulses of methanol. Regular samples were taken and methanol consumption was monitored using HPLC analysis (Figure 6). Changing the nitrogen source in the fed-batch experiment affected lactate production. Lactate production on urea as N source occurred more slowly. While the maximum lactate titer with ammonium sulfate as N source (2.6 ± 0.5 g/L, Figure 4A) was already reached after 9 days, a similar lactate titer of 2.6 ± 1.0 g/L was reached after 13 days using urea (Figure 6A). After these 13 days, lactate titers increased only minimally even with additional methanol pulses and reached a maximum value of 3.0 ± 1.0 g/L after 24 days. Thus, in contrast to cultivation with ammonium sulfate, no decrease in lactate titers was observed. It is further noticeable that when urea was used as N source, the pH did not decline during the cultivation, but increased from an initial value of 5.0 to a value of 5.7 ± 0.2 (Figure 6B). Also, the growth pattern was slightly changed with urea as N source. While again there is a long adaptation phase at the beginning of the cultivation (Figure 6A), growth significantly speeds up, after the first cultivation week, reaching a maximum between day 18 and 20 of the cultivation. However, little to no production was observed in this phase of maximal growth. Therefore, there seems to be a trade-off between lactate production and growth, as the highest lactate production rates were always obtained at the beginning of the cultivation when growth rates were low. In summary, the use of urea as N source did not lead to a drastically increased lactate titer compared to the original Verduyn medium and the rate of production even decreased. Under these conditions, it was observed that lactate titers did not decrease until the end of the cultivation. The yield of lactate per biomass between day 5 and 12 are promising, as with very little biomass lactate concentrations in the g/L range were produced.

### Adaptive laboratory evolution of lactate-producing strain

Although lactate production has already improved due to methanol feeding, the reproducibility of the obtained lactate titers was comparably low between the tested biological replicates (Figure 4; Figure 6). Additionally, the long cultivation time required, and the initial lag-phase are further disadvantages. The long cultivation time mainly results from the fact that methanol toxicity must be circumvented and only low amounts of methanol can be fed to the cultures at once. The Lh_LDH strain needs at least 5 days to consume the initial 0.5% methanol supplemented to the medium (Figure 4; Figure 6). Therefore, we evaluated whether cultivation times could be shortened by improving methanol tolerance and consumption in the Lh_LDH strain. To this end, an adaptive laboratory evolution (ALE) experiment was performed to improve growth on methanol. The strain overexpressing the Lh_LDH was cultivated on Verduyn medium with 2% methanol. Once the cells reached the exponential growth phase, they were transferred to fresh medium a total of 25 times. It must be noted that this approach carries the risk that lactate production of the Lh_LDH strain will cease completely in the evolved strains, as there is no direct selection pressure applied to guarantee lactate production remains active.

**FIGURE 7:**
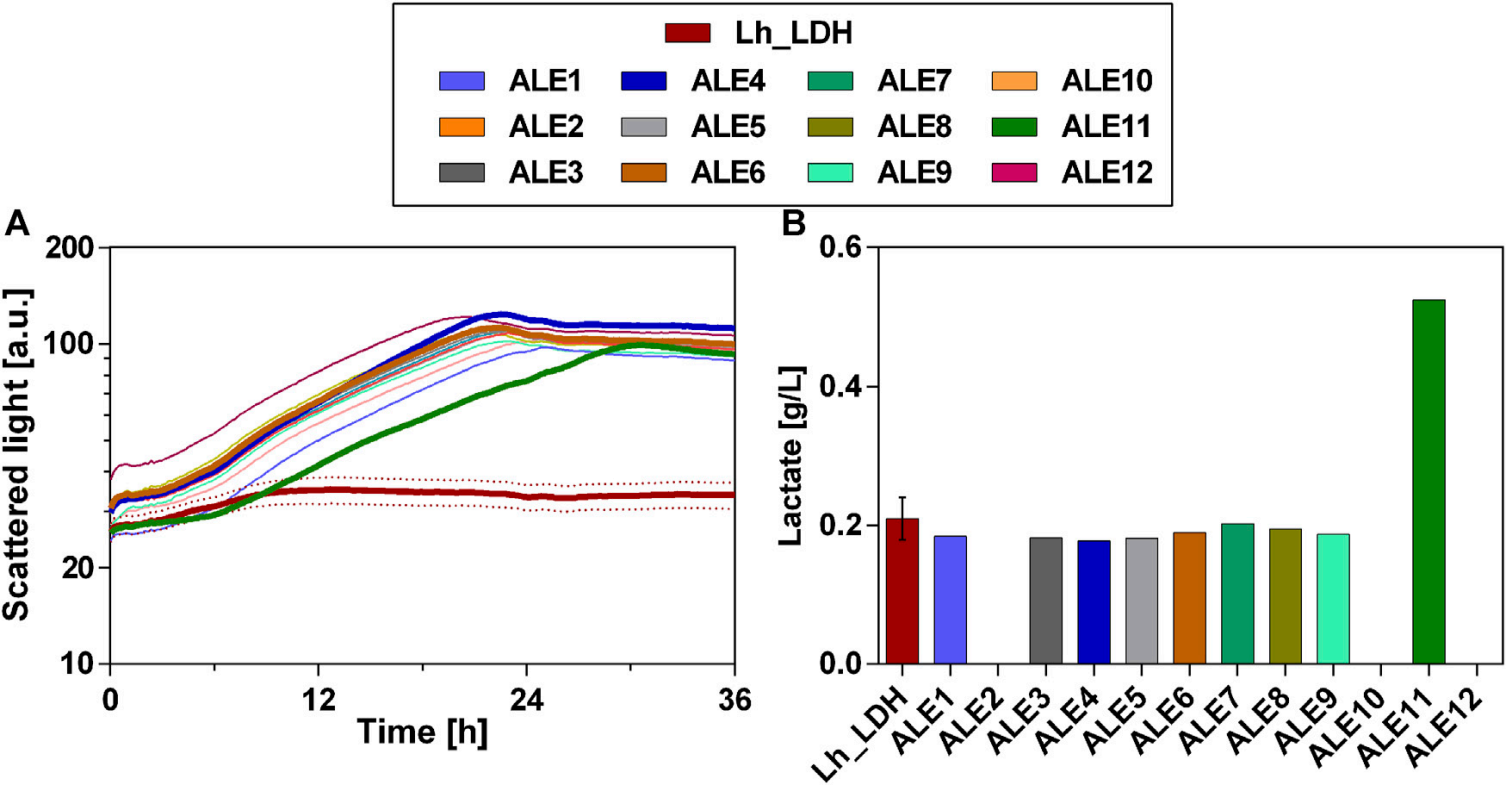
Growth and lactate production of single colonies of evolved *O. polymorpha* Lh_LDH population with methanol as carbon source. Development of biomass formation **(A)** and lactate titers at the end of the cultivation **(B).** The unevolved reference strain (Lh_LDH) was cultivated in biological triplicates, while the evolved colonies (ALE 1–12) were cultivated in single replicates. Cultivation in BioLector microtiter plates in Verduyn medium with 0.5% methanol as carbon source.

After the ALE experiment, the evolved populations were streaked out on YPD agar plates to obtain single colonies. 12 of these colonies were then cultivated in a microtiter plate experiment and compared to the original unevolved *O. polymorpha* Lh_LDH strain (Figure 7). All of the evolved colonies showed a significantly improved growth behavior, compared to the unevolved strain and already reached the stationary phase in the first 36 h of the experiment (Figure 7A). The unevolved Lh_LDH strain produced only 0.2 ± 0.0 g/L of lactate in this timeframe (Figure 7B). In eight of the evolved colonies tested, a similar lactate production level was achieved as in the unevolved strain. In three of the analyzed colonies, lactate production was even absent. However, for colony 11 lactate production increased compared to the unevolved strain and a lactate titer of 0.5 g/L was obtained (Figure 7B).

**FIGURE 8:**
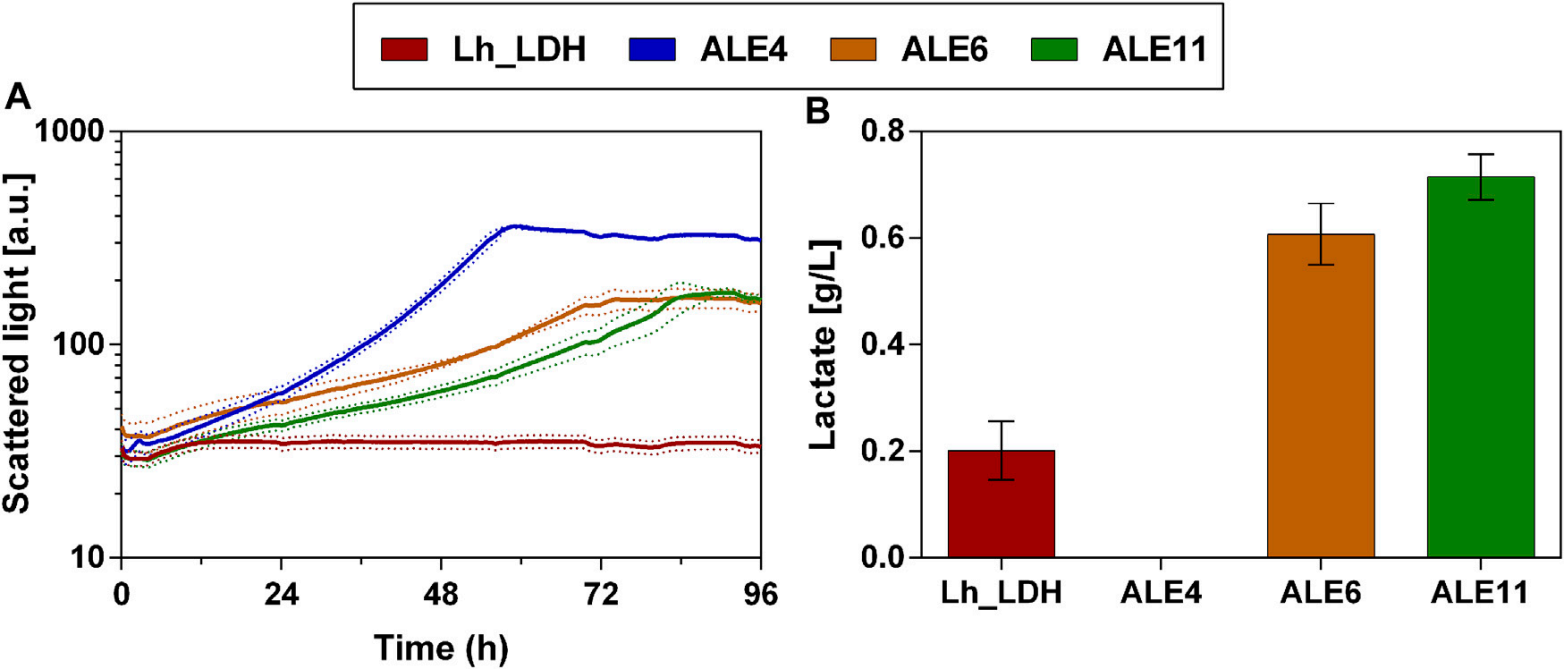
Growth and lactate production of selected evolved *O. polymorpha* colonies with 2% methanol as carbon source. Development of biomass formation **(A)** and lactate titers at the end of the cultivation **(B).** Cultivation in BioLector microtiter plates in Verduyn medium with 2% methanol as carbon source. Error bars represent the standard deviation from three biological triplicates.

Consequently, colony 11 was selected and compared again in biological triplicates to the unevolved strain (Figure 8). Additionally, the evolved colonies 4 and 6 were taken along as reference strains in this experiment, as they still showed lactate production and better growth than colony 11 in the preliminary screening of the colonies (Figure 7). These selected colonies were then cultivated on a higher methanol concentration of 2% and compared to the unevolved strain (Figure 8). This cultivation on 2% methanol as carbon source was performed with and without the addition of 1 g/L yeast extract to the medium. However, there was little difference in lactate titers and growth behavior for the ALE colonies between these two conditions (Cultivation without yeast extract: Figure 8; with additional yeast extract Supplementary Material: Supplementary Figure S5). Therefore, it was concluded that the evolved colonies can also be cultivated without the addition of yeast extract to the medium. Also with 2% methanol, the evolved strains showed drastically improved growth compared to the unevolved strain (Figure 8A). Particularly, colony 4 showed the highest growth rate and shortest lag-phase among the tested strains. However, when lactate titers were determined for that strain, no production was observed (Figure 8B). In contrast to that, colony 6 and 11 produced higher lactate levels than the unevolved strain (Colony 6: 0.6 ± 0.1 g/L; Colony 11: 0.7 ± 0.0 g/L). Again, colony 11 showed the highest lactate production of all tested colonies and was therefore chosen for further experiments. From now on colony 11 from this experiment will be referred to as ALE_Lh_LDH.

**FIGURE 9:**
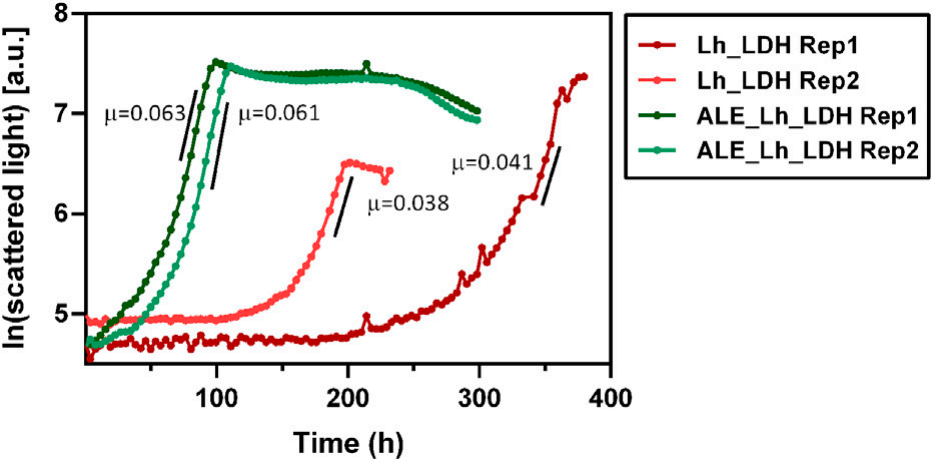
Growth curves of evolved *O. polymorpha* ALE_Lh_LDH strain (green) and the unevolved strain (Lh_LDH, red). Cultivation was performed in 500 mL shake flasks using 0.5% methanol as substrate and online biomass monitoring (scattered light). The *y*-axis shows the natural logarithm of the measured scattered light value. Maximum growth rates (µ) for each replicate (Rep) are given in h^-1^ next to the respective growth curve**.**

To compare their respective growth rates, the ALE_Lh_LDH and Lh_LDH strain were cultivated in 500 mL shake flasks equipped with the Cell Growth Quantifier (SBI Scientific Bioprocessing) to allow online biomass monitoring (Figure 9). Using 2% methanol as carbon source, the unevolved strain reached a maximum growth rate of 0.040 ± 0.002 h^-1^, while the evolved strain grew with a rate of 0.062 ± 0.001 h^-1^ (Figure 9). This corresponds to a 55% increase in growth rate. It further became clear that the evolved strain had a considerably reduced adaptation time to methanol as carbon source, which also increased the reproducibility of growth. The unevolved strain showed an unreproducible lag-phase differing from 100 to 200 h between replicates, which highlights that a concentration of 2% methanol already has a toxic effect on this strain. In contrast to that, the replicates for the ALE_Lh_LDH strain only had a short, reproducible lag-phase of less than a day.

To elucidate how the ALE_Lh_LDH strain can achieve high growth rates while at the same time keeping high levels of lactate production, this strain and its unevolved parental strain (Lh_LDH) were sent for whole genome sequencing. In comparison to the unevolved strain, there were six single-nucleotide polymorphisms (SNPs) found in the evolved strain. A summary of the identified mutations in the ALE_Lh_LDH strain can be found in Table 2.

**TABLE 2:** Single-nucleotide polymorphisms in the evolved *O. polymorpha* ALE_Lh_LDH strain compared to the unevolved Lh_LDH strain.

SNP No.	Nt position	Gene	Sequence change	Protein change
1	Contig 3, 213215	Methanol oxidase (MOX)	TAC → CAC	Tyr → His
2	Contig 4, 468138	Intergenic, Located between ORF for putative kynureninase & putative sulfate permease	G → A	
3	Contig 1, 575572	Downstream of LDH	G → A	
4	Contig 1, 575780	Downstream of LDH	AT → GC	
5	Contig 2, 1240617	Putative ORF	TGT → TCT	Cys → Ser
6	Contig 2, 1240654	Putative ORF	ATG → TTG	Met → Leu

The first SNP found (Table 2, SNP No.1) is located in the open reading frame (ORF) of the methanol oxidase (MOX) gene. The MOX catalyzes the first step of the methanol assimilation pathway, which is the oxidation of methanol to formaldehyde and hydrogen peroxide. The enzyme is located in the peroxisomes as a homo-octamer of 620 kDa (Shleev et al., 2006). The mutation changes a tyrosine residue in the methanol oxidase to a histidine residue (Y311H). This tyrosine residue is predicted to be part of a β-strand structure (AlphaFold Prediction). The tyrosine residue is conserved among the alcohol oxidases of methylotrophic yeast and forms part of the substrate binding domain in the *Komagataella phaffii* (*Pichia pastoris*) alcohol oxidase 1 (Vonck, Parcej, and Mills, 2016). This SNP consequently has a direct link to the observed phenotype of the ALE_Lh_LDH strain, as it could potentially explain why this strain is able to take up methanol considerably faster than the original unevolved strain. Further illustrations of this mutation can be found in the Supplementary Material in Section 9 (Supplementary Figure S9).

It is not immediately apparent whether any of the other mutations contribute to the observed phenotype. SNP No. 2 is located in an intergenic region located between the ORF for a putative kynureninase and a putative sulfate permease, neither of which appears to have an obvious association with the observed phenotype. SNP No. 3 and No. 4 are in close proximity to the inserted LDH gene, in a non-coding region 701 bp and 999 bp downstream of the LDH stop codon, respectively. Even though these SNPs most likely do not impact any expressed proteins, it might be conceivable that they affect the regulation of the LDH gene. SNP No. 5 and 6 are located in a predicted ORF and would lead to amino acid substitutions (Table 2). However, the predicted ORF would only code for a relatively small protein of 77 amino acids, and a BLASTp search against the non-redundant protein sequences database (nr), did not give any significant similarities. Therefore, it is questionable whether the predicted presence of an ORF at this location is accurate in this case.

### Performance of evolved strain in fed-batch cultivation

**FIGURE 10:**
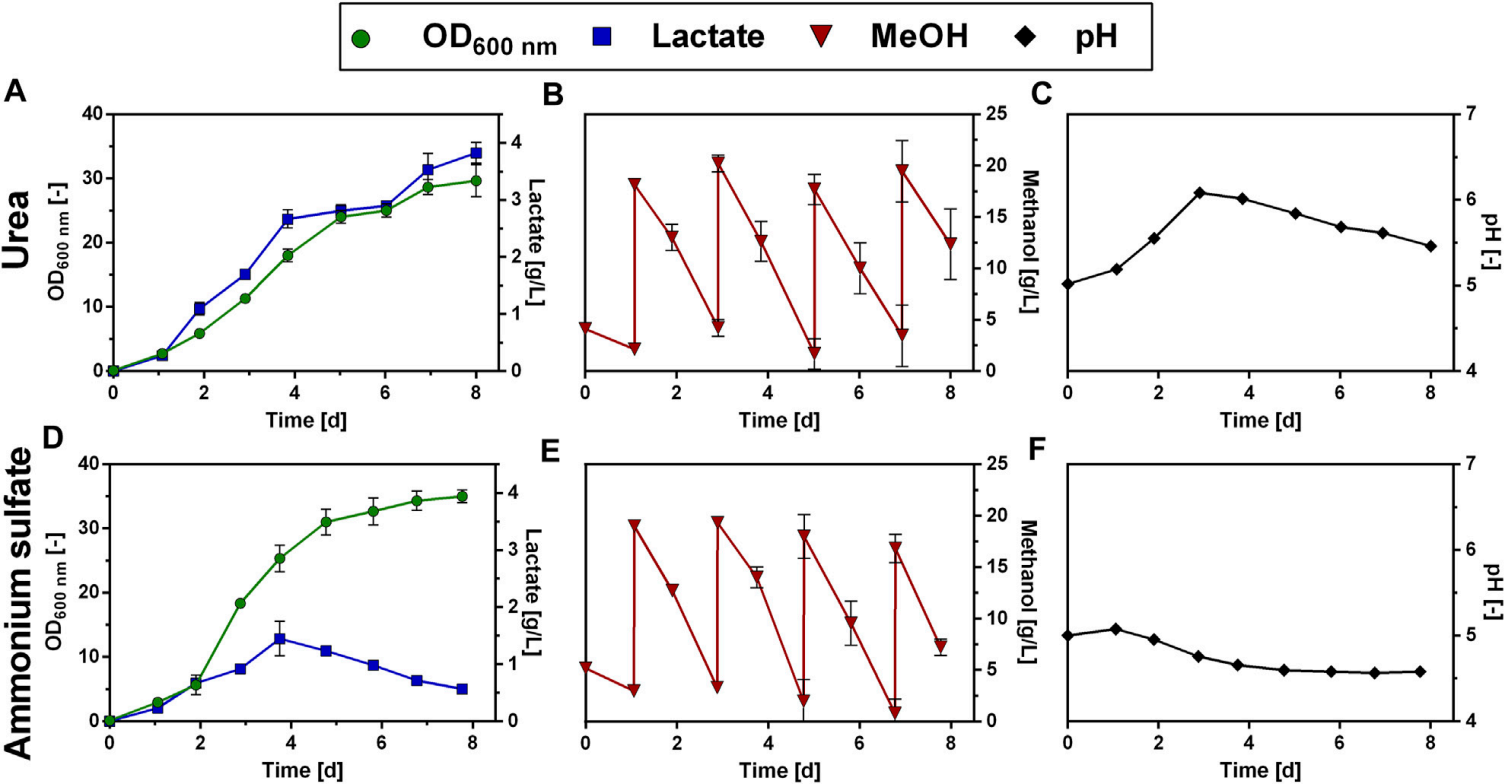
Methanol fed-batch with ALE_Lh_LDH strain. Cultivation of an evolved *O. polymorpha* strain overproducing an LDH of *Lactobacillus helveticus* using urea **(A,B,C)** or ammonium sulfate **(D,E,F)** as N source in the cultivation medium. Development of lactate titer (**A, D**; blue squares), optical density (**A, D**, OD_600 nm_, green dots), methanol concentration (**B, E**, red dots) and pH (**C, F**, black dots). Cultivations were done in Verduyn medium with 0.5% MeOH as initial carbon source. Additional pulses of 0.5% or 2% methanol were supplied to the culture. Experiments were performed in 500 mL shake flasks. Error bars represent the standard deviation of three biological replicates.

In order to analyze whether lactate titers and production rates could be improved with the ALE_Lh_LDH strain in a fed-batch setting, this strain was cultivated on Verduyn medium in 500 mL shake flasks, testing both ammonium sulfate and urea as N sources. As an initial starting concentration, 0.5% methanol was chosen to enable induction of LDH expression. Afterward, the cultures were fed with 2% of methanol upon methanol depletion. For the cultivation using urea as N source, it should be noted that the buffer in the Verduyn medium was changed from KH-phthalate to MES buffer (*cf. Methods* section). This was done, because very low lactate production was obtained when the ALE_Lh_LDH strain was cultivated with urea in a methanol fed-batch experiment (Supplementary Material: Supplementary Figures S6A,B). After an experiment time of 24 days and 6 consecutive methanol pulses of 2%, a lactate titer of only 1.05 ± 0.16 g/L was reached. It was noted that the pH rose drastically during the first 6 days of the experiment and reached a maximum value of 6.7 ± 0.0 at day 6, after which pH levels dropped again (Supplementary Material: Supplementary Figures S6C). This pH peak is outside the optimal pH range for *O. polymorpha* and was hypothesized to be the cause of the observed decrease in lactate production. In contrast to KH-phthalate which has a pK_a_ of 5.4, MES is capable of buffering in a higher pH range (pK_a_ 6.2). With this modified medium the fed-batch experiment was repeated (Figure 10). As expected, the ALE_Lh_LDH strain also grew substantially better in a fed-batch experiment compared to the unevolved strains. Again, growth differed dependent on the used N source. While the ALE_Lh_LDH strain with urea as N source reached a final optical density of 29.7 ± 2.5 within the 8 days of the experiment, it reached a higher biomass density of 31.0 ± 2.0 after only 5 days with ammonium as N source (Figures 10A,D). Apart from that, lactate production also differed considerably between the two different N sources. Using the MES buffer, it was possible to mitigate the pH increase for the ALE_Lh_LDH strain during the initial cultivation phase using urea as N source. The pH level never reached a value of above 6 (Figure 10C). As a result, a steady lactate production was observed. Already during the first 4 days, the lactate titer reached 2.7 ± 0.2 g/L (Figure 10A). In the methanol fed-batch experiment with the unevolved strain and urea as N source, a comparable titer (2.6 ± 1.0 g/L, Figure 6A) was reached only after 13 days of cultivation. For the ALE_Lh_LDH strain, the lactate titer continued to increase until the end of the experiment on day 8, but at a slightly lower rate, reaching a value of 3.8 ± 0.2 g/L on day 8 of the experiment. This was the highest titer observed so far in any of the experiments conducted. In contrast, cultivation of the ALE_Lh_LDH strain with ammonium sulfate as N source had no such positive effect on lactate production. After 4 days of cultivation, a lactate titer of only 1.5 ± 0.3 g/L was obtained (Figure 10D). After this, the lactate titer decreased again to a value of 0.6 ± 0.0 g/L on day 8 at the end of the cultivation. Thus, the ALE_Lh_LDH strain performed even worse than the unevolved Lh_LDH strain, which in 8 days reached a lactate titer of 2.6 ± 0.5 g/L (Figure 4A).

It should be emphasized that in these fed-batch experiments with the ALE_Lh_LDH, cells were supplied with pulses of 2% methanol (Figures 10B,E). Thus, the total amount of methanol being fed to the cells is higher compared to the previous fed-batch experiments with the unevolved strain (*cf.* Figure 4; Figure 6). To exclude that the increased lactate production simply resulted from the higher amount of methanol being fed to the evolved strain, the same experiment was repeated with the unevolved parental strain and a similar feeding pattern, in which after an initial pulse of 0.5% methanol, the cells were fed again with 2% methanol (Supplementary Material: Supplementary Figures S7). However, during the experimental time of 8 days, the unevolved strain was unable to consume the amount of methanol fed, only showed minimal growth, and produced only 0.5 g/L of lactate. Consequently, this demonstrates the increased ability of the evolved strain to tolerate and utilize higher concentrations of methanol, and thus reach higher lactate titers, which is not possible with the unevolved strain.

A summary of the obtained lactate titers, rates and production yields in the performed experiments can be found in Table 3. When calculating the lactate yield on methanol, it must be considered that considerable methanol evaporation occurs at 37 °C cultivation temperature. In an abiotic methanol evaporation experiment in the same cultivation system with 0.5% initial methanol concentration, half of the methanol had evaporated after about 4 days (Supplementary Material, Supplementary Figure S8). This methanol evaporation rate is significantly lower than the biological methanol uptake from the medium by *O. polymorpha* (app. 10 g/L/d for strain ALE_Lh_LDH, compare Figures 10B,E). However, the evaporation does not behave linearly and also varies to some extent between replicates (Supplementary Material: Supplementary Figures S8), so that the amount of evaporated methanol could not be determined precisely in the fed-batch experiments performed. Therefore, the reported yields were calculated based on the total amount of methanol fed and should be considered as an approximation and underestimation of the actual value.

**TABLE 3:** Maximum lactate production titers, rates and yields in all performed experiments. It is indicated in parentheses when the respective maximum value was reached during the culivation.

Strain	Cultivation mode	Max. Lactate titer (g/L)	Max. Production rate (g/L/d)	Max. Yield (approximate) (g_Lactate_/g_MeOH_)	Max. Specific yield (g_Lactate_/g_Biomass_)
Lh_LDH	Batch 0.5% methanol	0.5	-	0.12	1.62
Lh_LDH	Methanol fed batch + Ammonium	2.6 (Day 9)	0.29 (Day 9)	0.21 (Day 9)	1.59 (Day7)
Lh_LDH	Methanol fed batch + Urea	3.0 (Day 23)	0.20 (Day 13)	0.22 (Day 13)	2.44 (Day 13)
ALE_Lh_LDH	Methanol fed batch + Ammonium	1.5 (Day 4)	0.39 (Day 4)	0.06 (Day 1)	0.54 (Day2)
ALE_Lh_LDH	Methanol fed batch + Urea	3.8 (Day 8)	0.69 (Day 4)	0.08 (Day 3)	0.81 (Day 2)

While the ALE_Lh_LDH strain showed reproducible growth and lactate production on methanol, specifically with urea as N source, it has to be considered that it also consumes methanol faster and thus needs to be fed with higher amounts of methanol compared to the unevolved strain. Thus, the highest production titer and rate was achieved while cultivating the ALE_Lh_LDH strain with urea, but the production yield only reached 0.08 g_Lactate_/g_MeOH_ (Table 3). In comparison, the highest yield of 0.22 g_Lactate_/g_MeOH_ was obtained by the unevolved Lh_LDH strain with urea as N source (Table 3). This corresponds to 24% of the maximum theoretical yield (0.92 g_Lactate_/g_MeOH_). Further, it is noteworthy that the unevolved strain showed higher specific production per biomass compared to the evolved strain, reflecting that the increased amount of assimilated methanol in the ALE_Lh_LDH strain is not proportionally channeled into the product lactate.

## Discussion

In this study, the production of lactate from methanol using the yeast *O. polymorpha* was demonstrated. Further, the impact of methanol feeding, different N sources, and lactate transport were examined, and it was attempted to improve lactate production through adaptive laboratory evolution. While the highest lactate titers and production rates were achieved by using the evolved ALE_Lh_LDH strain in a methanol fed-batch experiment with urea as N source, the highest yields were obtained by the unevolved parental strain in a methanol fed-batch with urea as N source. These results show, how lactate production in the constructed strains is impacted by the medium composition, pH, and growth rates and indicate how further optimizations of the cultivation could be achieved.

The choice of N source was critical in the performed cultivations. In batch cultures with the same amount of total nitrogen, *O. polymorpha* produced different amounts of lactate with different N sources. In *Pichia pastoris*, it has been shown that the choice of N source can regulate the activity of genes involved in methanol assimilation (Rumjantsev et al., 2014). For example, the activity of the AOX1 promoter in *P. pastoris* was reported to be reduced in the presence of free amino acids in the cultivation medium (Velastegui et al., 2019). The expression of the LDH in this study is regulated by the MOX promoter, which is *O. polymorpha*’s native homolog to the AOX1 promoter. It is conceivable that the MOX promoter is similarly regulated by the N source, which could explain why yeast extract and peptone, which are N sources high in free amino acids, resulted in lower lactate titers compared to ammonium. However, this does not explain the increased lactate titers observed, when urea is used as N source. In yeasts, urea is converted to ammonia and CO_2_ by the urea amidolyase in an ATP-consuming reaction (Navarathna et al., 2010). Urea assimilation is consequently more energy-intensive than ammonium assimilation. Thus, it can be hypothesized that using urea as an N source leads to slower biomass formation, allowing more carbon to be channeled into lactate formation. This agrees well with the observation that the ALE_Lh_LDH strain grew more slowly and produced more lactate with urea as N source, and that for both the Lh_LDH and the ALE_Lh_LDH strain the highest lactate production rates were observed at lower growth rates. The fact that assimilation of urea is more energy-intensive than that of ammonium may also explain why the evolved ALE_Lh_LDH strain shows significantly better performance with urea as N source, while the non-evolved strain performs similarly with both ammonium and urea. The ALE_Lh_LDH strain mainly differs from the Lh_LDH strain in its ability to take up and utilize methanol. In the ALE_Lh_LDH strain methanol is assimilated much faster, leaving plenty of carbon available for both biomass and product formation. Since urea only allows slower biomass formation, more carbon can be used for lactate synthesis. In the unevolved Lh_LDH strain, however, lactate production instead seems to be limited by the methanol uptake rate, which is why the use of urea does not have such a positive impact on lactate production.

Additionally, another obvious factor influenced by the N source is the pH of the cultivation medium. It was observed that lactate production seems to be pH dependent in the created *O. polymorpha* strains. When urea was used as N source with KH-phthalate as a buffer, the pH increased drastically from an initial value of 5 to nearly 7 during the first cultivation days. While cell growth was still observed under these conditions, lactate production was almost completely abolished. It was reported that in ethanol fermentation of *S. cerevisiae* the use of urea as N source increases the intracellular pH of the yeast compared to ammonium (Yang et al., 2021). Since the LDH of *Lactobacillus helveticus* has a pH optimum of 5 (Savijoki and Palva, 1997), it is possible that the intracellular pH was outside the optimal range for lactate production, which might explain the pH sensitivity of lactate production in *O. polymorpha*.

It was further observed that when ammonium was used as the N source in fed-batch experiments, the lactate titers in the medium decreased again after a certain time point in the cultivation. Since this was not observed with urea as N source, it could potentially also be attributed to different pH levels. Whereas growth on urea initially increases the pH, the use of ammonium as an N source acidifies the cultivation medium. This is due to the fact that ammonium uptake in yeasts is proton-coupled, with a proton being released into the medium (Hensing et al., 1995). When the pH of the cultivation medium decreases, a larger fraction of the produced lactate will be present in its undissociated, uncharged form. As the undissociated lactate is more hydrophobic it can more easily cross the cell membrane by passive diffusion and thus be consumed again by the cells. This could explain why *O. polymorpha* does not consume externally supplied lactate in batch cultivations, where the pH does not drop drastically, but reabsorbs lactate in the fed-batch cultivations with ammonium where the pH decreases significantly. Consequently, it seems to be disadvantageous to perform lactate production with *O. polymorpha* with the developed strains at low pH levels. However, in recent years, many efforts have been made to carry out lactate fermentations at low pH values (Ghaffar et al., 2014; Park et al., 2018), as this can reduce the addition of neutralizing agents and thus greatly facilitate further downstream processing, specifically on an industrial production scale (Park et al., 2018). While *O. polymorpha* could potentially be an ideal host organism for this, due to its tolerance of low pH levels, in this study the highest titers were achieved with urea as N source, resulting in an increase in pH during the initial phase of cultivation and a slow decline thereafter. In the cultivation of the ALE_Lh_LDH strain with urea the final pH after 8 days of cultivation is still higher than the initial pH of 5, and well above the pK_a_ value of lactate at 3.86. While lactate production in *O. polymorpha* with ammonium as N source led to lower pH levels, it also resulted in the reuptake of lactate. Therefore, further optimization of lactate-producing strains and cultivation conditions is imperative. These optimizations should include culturing the strains in a more controlled environment. In this study, it was shown that both pH and methanol toxicity are critical factors for lactate production. Both can be regulated in a methanol fed-batch fermentation in a bioreactor, which then has the potential to further enhance lactate production. Additionally, it could be interesting to exploit the fact that increased lactate production occurs at lower growth rates. By limiting growth, e.g., through secondary substrate limitations, it might be possible to reroute the metabolic flux from biomass accumulation toward lactate production. Additionally, the fact that the ALE_Lh_LDH strain is able to assimilate methanol much faster could be exploited in the future. Further strain engineering could make it possible that this increased assimilation rate is not rerouted into growth and CO_2_ production but into lactate production.

This study thus provides a solid basis for further approaches for improving lactate production in *O. polymorpha* and contributes to the limited knowledge available on lactate production in methylotrophic yeasts. Yamada et al. produced D-lactate from methanol in the methylotrophic yeast *Pichia pastoris* and reached titers of 3.48 g/L of D-lactate (Yamada et al., 2019). The maximum achieved titer in this study, were 3.8 g/L of L-Lactate. Therefore, the production of both lactate enantiomers in methylotrophic yeasts from methanol has been demonstrated now. Recently, Baumschabl et al. produced up to 0.6 g/L of lactate from CO_2_ in an engineered autotrophic *Pichia pastoris* strain (Baumschabl et al., 2022). Their study points to a potential future, in which direct manufacturing of platform chemicals is possible from CO_2_ without the need for conversion to other C1 compounds (such as methanol). However, only low titers and slow growth rates can currently be achieved, and the system is still dependent on the addition of methanol for energy generation and promoter induction. Compared to CO_2_, methanol further has the advantage of being easily stored and transported. This could make it a key molecule for the storage and distribution of green energy, and indeed a methanol economy has previously been proposed (Olah, 2005).

Apart from insights into lactate production, this study also gives some information on the methanol metabolism of *O. polymorpha*. In the ALE_Lh_LDH strain, a SNP was identified, which leads to an amino acid substitution in the substrate binding domain of the methanol oxidase. Unsurprisingly, this result implies that the methanol oxidase plays an indispensable role in methanol assimilation. So far, there have been very few studies that examine the relationship between amino acid sequence and functionality of the MOX. While the C-terminal domain of the methanol oxidase is essential for the binding of the cofactor FAD and the targeting of the enzyme to the peroxisome (Gunkel et al., 2004), no mutations in the central substrate binding domain have been analyzed so far. It should still be clarified via reverse engineering whether the observed amino acid substitution in the MOX is exclusively responsible for the observed phenotype or if any of the other observed SNPs contribute to it. Nonetheless, this result suggests that there might be a potential to increase the enzyme performance of the methanol oxidase and with that methanol assimilation efficiency of *O. polymorpha* through single amino acid substitutions. This could further push the use of methylotrophic yeasts for the industrial production of important platform chemicals from CO_2_-derived substrates such as methanol.

## Conclusion

This study shows how methylotrophic yeasts can potentially contribute to sustainable biomanufacturing based on C1 compounds. Through genetic engineering, medium adaptation, and adaptive laboratory evolution we were able to engineer a strain that shows improved and reproducible growth on methanol while at the same time producing lactate without a long adaptation phase. Therefore, this study provides guidance on how methylotrophic organisms could contribute to the future of a sustainable, low land-use, bioeconomy through the production of platform chemicals from CO_2_-derived substrates.

## Data Availability

The original contributions presented in the study are included in the article/Supplementary Material, further inquiries can be directed to the corresponding authors.
