# Supplementary material for: Engineering the methylotrophic yeast Ogataea polymorpha for lactate production from methanol
Source: Front Bioeng Biotechnol. 2023 Jun 30;11:1223726. doi: 10.3389/fbioe.2023.1223726 (PMC10347679; doi:10.3389/fbioe.2023.1223726)
Supplement: Supplementary file 1 [file DataSheet1.docx]

# Engineering the methylotrophic yeast *Ogataea polymorpha* for lactate production from methanol

Katrin Wefelmeier^1^, Simone Schmitz^1^, Anna M. Haut^1^, Johannes Otten^1^, Tobias Jülich^1^, Lars M. Blank^1*^

*1 iAMB - Institute of Applied Microbiology, ABBt – Aachen Biology and Biotechnology, RWTH Aachen University, Worringerweg 1, D-52074 Aachen, Germany*

*** *Correspondence:* *Lars.Blank@rwth-aachen.de*

# Supplementary Information

## End pH values after microtiter plate batch cultivation of LDH strains on methanol

|   **Figure S1 –** **pH levels at the end of a cultivation for O. polymorpha strains overexpressing lactate dehydrogenase genes** of Bos taurus, Leuconostoc mesenteroides, Lactobacillus helveticus or Lactiplantibacillus plantarum. Cultivations were performed in a microtiter plate cultivation experiment with **(A)** 0.5 % (v/v) methanol (MeOH) or **(B)** 2 % (v/v) MeOH as carbon source and lactate titers and end OD were measured after a cultivation time of 7 days. Cultivation of Lh_LDH strain on lactate and methanol   **Figure S2** – **Cultivation of the O. polymorpha Lh_LDH strain with 0.5 % methanol (v/v) and 2.5 g/L or 5 g/L of L-lactate** supplied to the medium. Cultivation was performed in a BioLector microtiter plate. Final lactate and methanol titers were measured after a cultivation time of 7 days. Error bars represent the standard deviation of three biological replicates. Testing glucose and glycerol as co-substrates   **Figure S3 - Lactate production (C) and optical density (OD, B) of O. polymorpha strains overexpressing a lactate dehydrogenase gene from Lactobacillus helveticus**. Cultivations were performed in a microtiter plate cultivation experiment with the indicated **(B, C;** x-axis) combination of the carbon sources glucose, glycerol, and methanol. Methanol was either added during the exponential growth phase of a culture **(+ Methanol)** or already supplied at the beginning of the cultivation **(& Methanol)**. Panel **A** shows an exemplary growth curve in which O. polymorpha was grown on 15 g/L of glycerol and 0.5 % (v/v) were added to the culture (dotted line) during exponential growth phase. Fed-batch cultivation of Bt_LDH strain In this fed-batch experiment the Bt_LDH strain was initially fed with 0.5% methanol. As the strain showed no growth during the initial 6 days and consumed the initially supplied 0.5% methanol with a much slower rate compared to the Lh_LDH strain, the strain was afterwards fed with even lower pulses of 0.125 % methanol, to avoid methanol toxicity. Once considerable growth was observed the cells were again fed with 0.5 % methanol.    **Figure S4 -** **Cultivation of O. polymorpha Bt_LDH in a methanol fed batch experiment.**  Development of lactate titer (blue squares), optical density (OD_600 nm_, green dots), cultivating an O. polymorpha strain overexpressing Bt_LDH cultivated in Verduyn medium 0.5 % MeOH as initial carbon source. Additional pulses of 0.5 % or 0.125% methanol (red and black triangles, respectively) were supplied to the culture upon methanol consumption. Experiments were performed in 500 mL shake flasks. Error bars represent the standard deviation of three biological replicates. Testing ALEd colonies on 2 % methanol and 1 g/L yeast extract   **Figure S5 - Growth and lactate production of selected evolved O. polymorpha colonies with 2 % methanol as carbon source and 1 g/L of supplemented yeast extract.** Comparison of the unevolved Lh_LDH strain (WT) and the evolved colonies (K4, K6, K11). Development of biomass formation (**A**) and lactate titers at the end of the cultivation (**B**)**.** Cultivation in BioLector microtiter plates in Verduyn medium with 2 % methanol as carbon source. Error bars represent the standard deviation from three biological triplicates. |  |  |  |  |  |  |  |  |  |  |  |  |  |  |  |  |
| --- | --- | --- | --- | --- | --- | --- | --- | --- | --- | --- | --- | --- | --- | --- | --- | --- |
| Fed-batch of ALE_Lh_LDH strain with urea as N-source   **Figure S6 - Methanol fed batch of ALE_Lh_LDH strain on Verduyn medium with KH-phthalate buffer and urea as N source.** Development of lactate titer (**A**, blue squares), optical density (**A**, OD600, green dots), methanol concentration (**B**, red dots) and pH (**C**, black dots) cultivating an evolved O. polymorpha strain overexpressing Lh_LDH cultivated in Verduyn medium with urea as N source and 0.5 % MeOH as initial carbon source. Additional pulses of 0.5 % or 2 % methanol were supplied to the culture upon methanol consumption. Experiments were performed in 500 mL shake flasks. Error bars represent the standard deviation of three biological replicates. Fed-batch of Lh_LDH strain with methanol pulses of 2 %   **Figure S7 - Methanol fed batch of unevolved Lh_LDH strain on Verduyn medium with urea as N source.** Development of lactate titer (**A**, blue squares), optical density (**A**, OD600, green dots), methanol concentration (**B**, red dots) and pH (**C**, black dots) cultivating an evolved O. polymorpha strain overexpressing Lh_LDH cultivated in Verduyn medium with urea as N source and 0.5 % MeOH as initial carbon source. An additional pulse of 2 % methanol was supplied to the culture upon methanol consumption. Experiments were performed in 500 mL shake flasks. Error bars represent the standard deviation of three biological replicates. Methanol evaporation in shake flasks   ***Figure S8 – Methanol Evaporation*** *in 500 mL shake flasks, filled with 50 mL water and a starting concentration of 0.5% methanol. Cultivation at 37 °C and 250 rpm shaking. Error bars represent the standard deviation of 3 replicates.* |  |  |  |  |  |  |  |  |  |  |  |  |  |  |  |  |

## Point mutation of methanol oxidase in evolved *O. polymorpha* strain

The following section shows a multiple sequence alignment of alcohol oxidases of methylotrophic yeast as well as structural prediction of the methanol oxidase of *O. polymorpha*. The point mutation observed in the ALE_Lh_LDh strain is highlighted.

The multiple sequence alignment below was created with ClustalW of alcohol oxidases of methylotrophic yeast. Alcohol oxidase sequences homologous to the methanol oxidase of *Ogataea polymorpha* from the following yeast species were chosen: *Candida boidinii*, *Kuraishia capsulata* and *Komagataella phaffi* (*Pichia pastoris*). The tyrosine residue that is mutated to a histidine residue in the ALE_Lh_LDH strain is shown in red letters. Residues belonging to the substrate binding domain are highlighted in gray (according to Vonck et al., 2016). Figure S9 shows the location of the mutated tyrosine residue as part of a beta-strand structure. The structural predictions were made with the AlphaFold Monomer v2.0 pipeline (Varadi et al. 2022; Jumper et al. 2021).

**Ogataea MAIPDEFDIIVVGGGSTGCCIAGRLANLDDQNLTVALIEGGENNINNPWVYLPGVYPRNM 60**

Candida MAIPEEFDVIVCGGGSTGCVIAGRLANVDE-NLKVLLIENGENNLNNPWVYLPGIYPRNM 59

Komagataella MAIPEEFDILVLGGGSSGSCIAGRLANLDH-SLKVGLIEAGENNLNNPWVYLPGIYPRNM 59

Kuraishia MAIPDEFDIIVAGGGSAGCCIAGRLANLDP-ELKVCLIEAGENNLNNPWVYLPGIYPRNM 59

****:***::* ****:*. *******:* .*.* *** ****:*********:*****

**Ogataea RLDSKTATFYSSRPSKALNGRRAIVPCANILGGGSSINFLMYTRASASDYDDWESEGWST 120**

Candida RLDSKTATFYNSRPSKHLNGRRAIVPQANILGGGSSINFMMYTRASASDYDDWESEGWTT 119

Komagataella KLDSKTASFYTSNPSPHLNGRRAIVPCANVLGGGSSINFMMYTRGSASDYDDFQAEGWKT 119

Kuraishia QLDSKTATFYTAEPSPHLNGRRAIVPCANILGGGSSINFMMYTRGSASDYDDWETEGWTT 119

:******:**.:.** ********* **:*********:****.*******:::***.*

**Ogataea DELLPLIKKIETYQRPCNNRDLHGFDGPIKVSFGNYTYPTCQDFLRAAESQGIPVVDDLE 180**

Candida DELLPLMKKFETYQRPCNNRDLHGFDGPIKVSFGNYTYPQCQDFLRACETQGIPYVDDLE 179

Komagataella KDLLPLMKKTETYQRACNNPDIHGFEGPIKVSFGNYTYPVCQDFLRASESQGIPYVDDLE 179

Kuraishia KDLLPLMKKTETYQRACNNRDIHGFEGPIKVSFGNYTYDNCQDFLRAAESQGIPVVDDLE 179

.:****:** ***** *** *:***:************ *******.*:**** *****

**Ogataea DFKTSHGAEHWLKWINRDLGRRSDSAHAYVHPTMRNKQSLFLITSTKCDKVIIEDGKAVA 240**

Candida DLTTSHGAEQWLKWINRDLGRRSDTAHAFIHSTMRNKENLFLLTNTKVDKVIIENGRAVA 239

Komagataella DLVTAHGAEHWLKWINRDTGRRSDSAHAFVHSTMRNHDNLYLICNTKVDKIIVEDGRAAA 239

Kuraishia DLVTAHGAEHWLKWINRDTGRRSDSAHAFIHSTMRNKDNLFLVTSTKIDKIIVEDGRAVG 239

*: *:****:******** *****:***::* ****::.*:*: .** **:*:*:*:*..

**Ogataea VRTVPMKPLNPKKPVSRTFRARKQIVISCGTISSPLVLQRSGIGAAHHLRSVGVKPIVDL 300**

Candida VRTVPSKPIGDSK-VSRTFKARKQIVVSCGTISSPMVLQRSGIGEPSKLRAAGVKPIVEV 298

Komagataella VRTVPSKPLNPKKPSHKIYRARKQIVLSCGTISSPLVLQRSGFGDPIKLRAAGVKPLVNL 299

Kuraishia VKTVPSKPLDPKNPKTRTFRARKQIVLSCGTVSSPMVLQRSGFGDPQKLRAAGVKPLVNL 299

*:*** **:. .: : ::******:****:***:******:* :**:.****:*::

**Ogataea PGVGENFQDHYCFFTPYYVKPDVPTFDDFVRGDPVAQKAAFDQWYSNKDGPLTTNGIEAG 360**

Candida PGVGKNFQDHYCYFVPYRIKHDSESFDAFVSGDKEAQKSAFDQWYATGSGPLATNGIEAG 358

Komagataella PGVGRNFQDHYCFFSPYRIKPQYESFDDFVRGDAEIQKRVFDQWYANGTGPLATNGIEAG 359

Kuraishia PGVGRNFQDHYCFFTPYRIKPHLESFDDFVRGDKDVQKKVFDQWYANGTGPLATNGIEAG 359

****.*******:* ** :* . :** ** ** ** .*****:. ***:*******

**Ogataea VKIRPTEEELATADEDFRRGYAEYFENKPDKPLMHYSVISGFFGDHTKIPNGKFMTMFHF 420**

Candida VKIRPTQAELATADKGFQDGWETYFENKPDKPLMHYSVISGFFGDHTKLPPGKYMTMFHF 418

Komagataella VKIRPTPEELSQMDESFQEGYREYFEDKPDKPVMHYSIIAGFFGDHTKIPPGKYMTMFHF 419

Kuraishia VKIRPTEEELAAMDSDFREGYEEYFRNKPDKPVMHYSVISGFFGDHTKIAPGKYMSMFHF 419

****** **: *..*: *: **.:*****:****:*:********: **:*:****

**Ogataea LEYPFSRGFVRITSANPYDAPDFDPGFLNDERDLWPMVWAYKKSRETARRMESFAGEVTS 480**

Candida LEYPFSRGWLHISSDDPYDSPDFDPGFLNDDRDMWPMVWAFKKSRETARRMECFAGEPTP 478

Komagataella LEYPFSRGSIHITSPDPYAAPDFDPGFMNDERDMAPMVWAYKKSRETARRMDHFAGEVTS 479

Kuraishia LEYPFSRGYVQIKSADPYAAPTFDPGFMNDKRDMAPMIWAYKKSRETARRMDAFAGEVTS 479

******** ::*.* :** :* *****:**.**: **:**:**********: **** *

**Ogataea HHPLFKVDSPARARDLDLETCSAYAGPKHLTANLYHGSWTVPIDKPTPKNDFHVTSNQVQ 540**

Candida FHPHYPCDSPARALEQSLEDTRKLAGPLHLTASLYHGSWSTTIGEADKHNPSHVTSSHIN 538

Komagataella HHPLFPYSSEARALEMDLETSNAYGGPLNLSAGLAHGSWTQPLKKPTAKNEGHVTSNQVE 539

Kuraishia HHPYFPYDSDARALEMDLETSKAYGGPLHLSANLAHGSWSQPIGKPSIKNADHCTSNQVG 539

.** : .* *** : .** .** :*:*.* ****: : : :* * **.::

**Ogataea L-HSDIEYTEEDDEAIVNYIKEHTETTWHCLGTCSMAPREGSKIAPKGGVLDARLNVYGV 599**

Candida VYGKNIQYTEDDDKAIEKYIKEHVETTWHCLGTNSMAPLEGNKNAPEGGVLDPRLNVHGV 598

Komagataella L-HPDIEYDEEDDKAIENYIREHTETTWHCLGTCSIGPREGSKIVKWGGVLDHRSNVYGV 598

Kuraishia L-HAEIEYTKEDDLAIENYIKEHTETTWHCLGTNSMAPREGSKIAPHGGVLDPRANVYGV 598

: :*:* ::** ** :**:**.********* *:.* **.* . ***** * **:**

**Ogataea QNLKVADLSVCPDNVGCNTYSTALTIGEKAATLVAEDLGYSGSDLDMTIPNFRLGTYEET 659**

Candida KGLKVADLSVCPDNVGCNTFSTALTIGEKAAVLIAEDLGYSGSALDMEVPQFKLKTYEQS 658

Komagataella KGLKVGDLSVCPDNVGCNTYTTALLIGEKTATLVGEDLGYSGEALDMTVPQFKLGTYEKT 658

Kuraishia KGLKVGDLSVCPDNVGCNTYTTALLIGEKIATLVAEDLGYSGDDLAMEVPQFKLGTYEKT 658

:.***.*************::*** **** *.*:.*******. * * :*:*:* ***::

**Ogataea GLARF 664**

Candida GAARY 663

Komagataella GLARF 663

Kuraishia GLARF 663

* **:


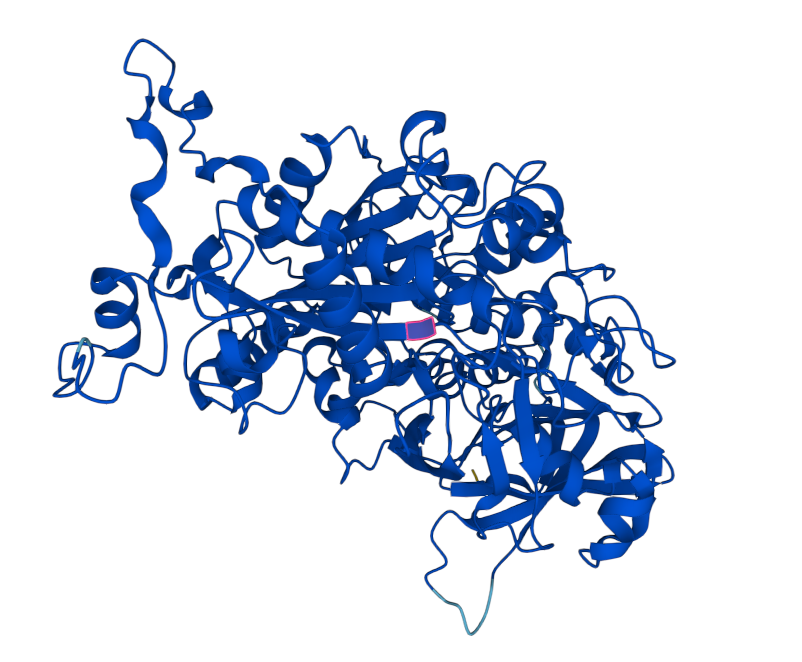

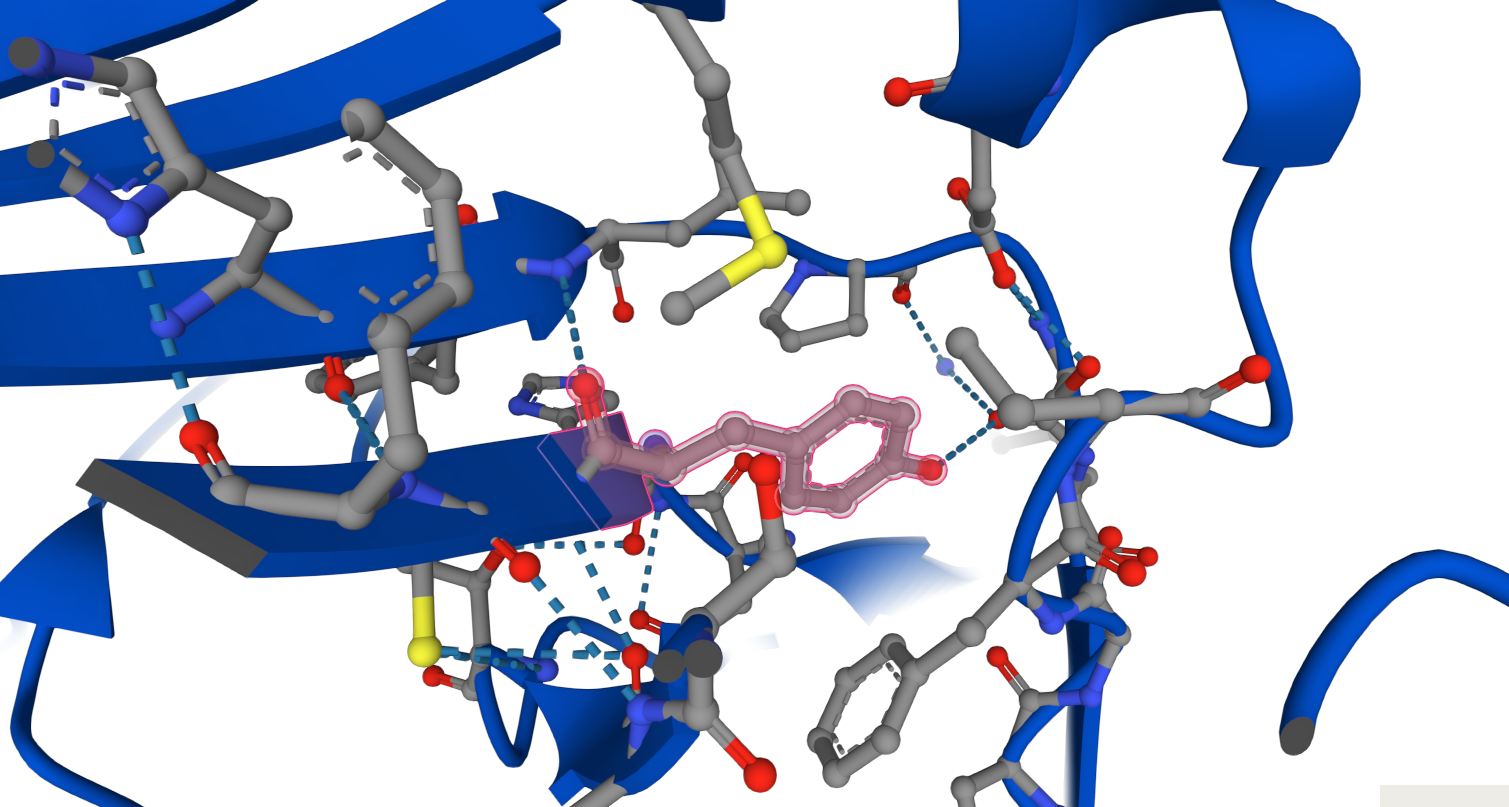


**A**

**B**

***Figure S9*** *– (****A****) Predicted location of mutated tyrosine residue (pink) in the methanol oxidase monomer of* O. polymorpha*. (****B****) Predicted interaction of mutated tyrosine residue (pink) with surrounding side chains. Structure predictions were made with the AlphaFold Monomer v2.0 pipeline.*

## References

Jumper, J., Evans, R., Pritzel, A., Green, T., Figurnov, M., Ronneberger, O., ... & Hassabis, D. (2021). Highly accurate protein structure prediction with AlphaFold. *Nature*, *596*(7873), 583-589.

Varadi, M., Anyango, S., Deshpande, M., Nair, S., Natassia, C., Yordanova, G., ... & Velankar, S. (2022). AlphaFold Protein Structure Database: massively expanding the structural coverage of protein-sequence space with high-accuracy models. *Nucleic acids research*, *50*(D1), D439-D444.

Vonck, J., Parcej, D. N., & Mills, D. J. (2016). Structure of alcohol oxidase from Pichia pastoris by cryo-electron microscopy. *PloS one*, *11*(7), e0159476.
